# HCP5 Derived Novel Microprotein Triggers Progression of Gastric Cancer through Regulating Ferroptosis

**DOI:** 10.1002/advs.202407012

**Published:** 2024-10-24

**Authors:** Qiuhui Li, Guoqing Guo, Yuli Chen, Lu Lu, Hanyang Li, Zihan Zhou, Jiahao Guo, Xiongkang Gan, Yanming Hu, Qiunuo Li, Ming Sun, Xianghua Liu

**Affiliations:** ^1^ Department of Biochemistry and Molecular Biology School of Basic Medical Sciences Nanjing Medical University Nanjing 211166 China; ^2^ Suzhou Cancer Center Core Laboratory The Affiliated Suzhou Hospital of Nanjing Medical University Suzhou Municipal Hospital Gusu School Nanjing Medical University Suzhou 215001 China; ^3^ The First Clinical Medical College Nanjing Medical University Nanjing 211166 China; ^4^ Department of Cardiovascular Medicine The First Affiliated Hospital of Nanjing Medical University Nanjing 210029 China

**Keywords:** ferroptosis, gastric cancer, HCP5‐132aa, m^5^C modification, YBX1

## Abstract

The context of long noncoding RNAs (lncRNAs) contains many unannotated open reading frames (ORFs). These ORFs potentially encode novel proteins or peptides with crucial roles in various human cancers, yet the translational potential of these lncRNAs and the functions of the protein products remain largely unexplored, especially in gastric cancer (GC). In this study, a comprehensive analysis is performed and identified a GC associated lncRNA known as HCP5, which contains a non‐canonical ORF. Further analysis showed that HCP5‐132aa, a microprotein encoded by HCP5 harboring this ORF, is highly expressed in GC cells and tissues, and can promote the proliferation of GC cells by inhibiting ferroptosis. Mechanistically, HCP5‐132aa enhances the interaction between YBX1 and ELAVL1, facilitates recognition of YBX1 at the m^5^C site in the 3′UTR of SLC7A11 and G6PD mRNA, and preserves their stability via ELAVL1. By employing a Cas9/sgRNA delivery system with AAV in vivo, effectively knocked out the HCP5‐132aa and inhibition of tumor growth in a patient‐derived xenograft model are achieved. These findings demonstrate that the novel protein HCP5‐132aa, derived from lncRNA HCP5, mediates the repression of ferroptosis, thereby driving the progression of GC and identifying a new potential therapeutic target for its treatment.

## Graphical abstract

This study describes the characterizes of a lncRNA HCP5‐derived microprotein, HCP5‐132aa, which exhibits an onco‐promotion effect and presents a prospective therapeutic target for the treatment of gastric cancer.

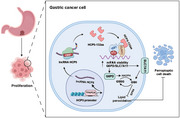

## 1 Introduction

Gastric cancer (GC) is one of the most common malignant tumors worldwide, and a major contributor to tumor‐related deaths.^[^ ^1^, ^2^ ^]^ According to statistical data from the National Cancer Center of China, GC ranks third in the country in both annual incidence and death rate, which is significantly higher than in other regions.^[^ ^3^, ^4^ ^]^ Even though molecular diagnostic technology, targeted therapy and immune checkpoint inhibition therapy have made great progress in recent years, the 5‐year overall survival rate of GC is still unsatisfactory, less than 30%.^[^ ^5^ ^]^ The main reason for this is that most patients with GC are already at a late stage of the disease when diagnosed due to the lack of effective molecular indicators that could facilitate early diagnosis. Therefore, a deeper understanding of the molecular mechanism underlying gastric carcinogenesis and progression is not only beneficial to provide potential molecular markers for early diagnosis but may also provide valuable targets for developing novel therapy strategy.

In recent years, the rapid advancement of translationomic sequencing represented by ribosome profiling (Riboseq), ribosome‐nascent chain complex‐seq (RNC‐seq), and protein mass spectrometry (MS) analysis technologies has enabled researchers to scrutinize RNA translation activities at a genome‐wide level.^[^ ^6^, ^7^ ^]^ Interestingly, a growing number of studies have shown that the context of long noncoding RNAs and circular RNAs contains many unannotated non‐classical open reading frames (ORFs), any of which could encode novel functional proteins or peptides.^[^ ^8^, ^9^, ^10^ ^]^ Golub and colleagues experimentally investigated 553 non‐canonical ORFs in lncRNAs and regions upstream or downstream of protein coding genes by integrative analyzing riboseq data and MS data. Among these ORFs, 227/553 (41%) were confirmed as translated and 57 induced viability defects after knockout in cancer cells with CRISPR/Cas9 tiling screen.^[^ ^11^ ^]^ In our previous study, we conducted an integrative analysis combining Riboseq and CRISPR/Cas9 screening, allowing us to identify 758 lncRNAs‐encoded ORFs commencing with the ATG start codon. By conducting an in‐depth analysis of CRISPR screen and The Cancer Genome Atlas (TCGA) data, we identified 28 functional ORFs. Subsequently, we determined that GT3‐INCP, which is encoded by LINC00992, plays an oncogenic role by interacting with the transcription factor GATA3 in breast cancer.^[^ ^12^ ^]^ These findings demonstrated that cryptic lncRNA or circRNA derived proteins could be important regulators involved in carcinogenesis and cancer progression, and indicated that these “hidden” proteins encoded by lncRNAs or circRNAs may be a new resource for therapeutic target discovery.

To date, only a few circRNA encoded proteins have been identified and characterized in GC. For instance, CM‐248aa encoded by circMTHFD2L, was found to suppress the proliferation and metastasis of GC cells by competitively targeting SET and inhibiting the SET‐PP2A interaction, thereby promoting the dephosphorylation of AKT and P65.^[^ ^13^ ^]^ CircAXIN1 derived novel protein AXIN1‐295aa triggers GC tumorigenesis and progression via activation of Wnt signaling pathway by interacting with APC, which leads to β‐catenin nucleus translocation and downstream gene transcription.^[^ ^14^ ^]^ As mentioned previously, there exists a substantial number of unannotated non‐classical ORFs within the context of lncRNAs. However, their potential functions and roles in GC remain to be elucidated. In this study, we conducted an in‐depth analysis by integrating ribosome Riboseq data, RNAseq data and microarray data from the TCGA and GEO to identify GC associated lncRNAs with protein encoding potential. We further determined the translation of a cryptic lncRNA HCP5‐encoded protein named HCP5‐132aa and characterized its biological function and underlying mechanism in GC tumorigenesis and progression.

## 2 Results

### 2.1 HCP5‐132aa Encoded by lncRNA HCP5 Exhibits a Pronounced Upregulation in GC

**Figure 1:**
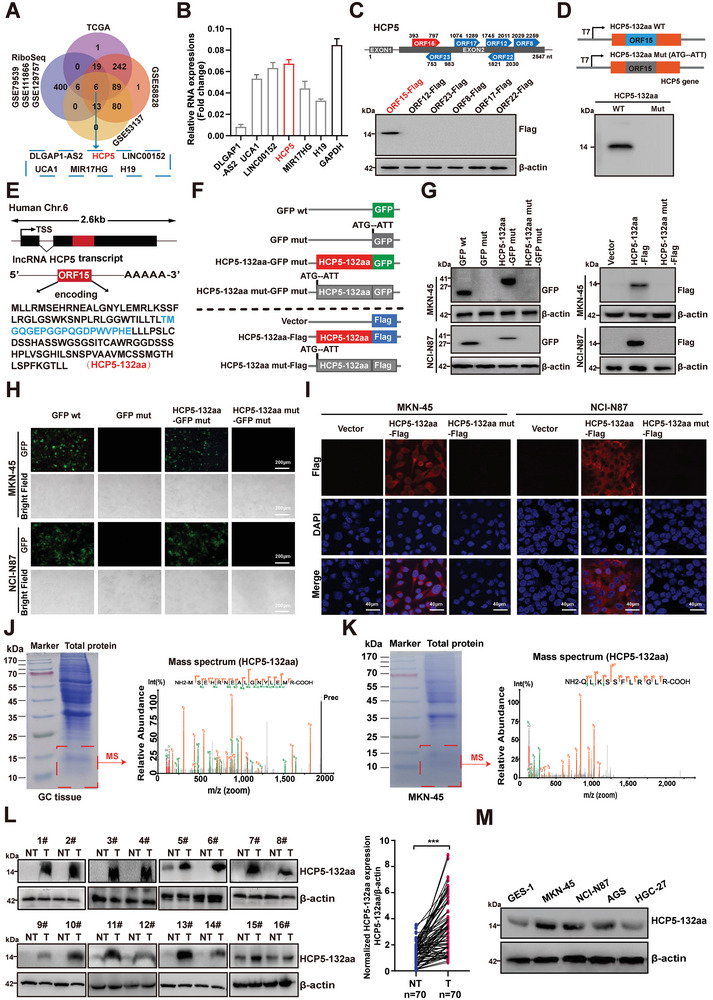
HCP5‐132aa encoded by the lncRNA HCP5 exhibits a pronounced upregulation in GC. A) The Venn diagram illustrates six lncRNAs with coding potential in GC, and the data was obtained from TCGA and GEO (GSE58828, GSE53137) as well as ribosome‐associated lncRNAs datasets (GSE79539, GSE111866, GSE129757). B) The enrichment of six candidate lncRNAs on polyribosomes in GC cells was analyzed using polysome profiling followed by RT‐qPCR (*n* = 3). Student's t‐test. C) The expression of each ORF‐Flag fusion protein was assessed by western blotting using anti‐Flag antibodies, following transfection of six ORF‐Flag fusion constructs into HEK‐293T cells for 48 h. D) Diagram of the constructs used for in vitro translation of the HCP5‐132aa microprotein. Cloning of full‐length lncRNA HCP5 sequences, comprising both wild‐type (WT) and ATG‐mutated variants with the T7 promoter, into the pcDNA3.1 vector was performed, followed by in vitro transcription and translation coupling to generate a microprotein of ≈14 kDa (HCP5‐132aa), which was visualized using SDS‐PAGE. E) Schematic diagram shows that ORF15 is located within exon 2 of the annotated lncRNA HCP5 in the human genome, which encodes a microprotein HCP5‐132aa. F) Diagram of the GFP and Flag fusion constructs. The start codon ATG of the GFP and HCP5‐132aa was mutated to ATT. G–I) Indicated constructs were transfected in GC cells, HCP5‐132aa‐GFP and HCP5‐132aa‐Flag fusion protein expression was detected by western blotting with anti‐GFP and Flag antibodies, respectively (G); the GFP fluorescence was observed using fluorescence microscope (H); Flag signal (red) was detected by immunofluorescence (I), Scale bar: 40 µm. J,K) After excising the differential gel bands in the vicinity of 15 kDa and subjecting them to MS analysis after Coomassie Brilliant Blue staining, the endogenous expression of HCP5‐132aa was identified in GC tissues and cells. L) Seventy pairs of GC tissues were examined for HCP5‐132aa protein expression through western blotting, utilizing customized anti‐ HCP5‐132aa antibodies (16 pairs shown, left). Paired t‐tests were used to compare expression levels of HCP5‐132aa protein in adjacent tissues and GC samples (right). M) HCP5‐132aa expression levels were measured in several established GC lines by western blotting. Data are representative of three independent experiments. GFP, green fluorescent protein; NT, non‐tumor; T, tumor. ***p < 0.001.

In the pursuit of identifying lncRNAs harboring protein‐coding potential in GC, a thorough analysis was conducted on diverse RNA sequencing datasets, including lncRNA expression profiles pertaining to GC from TCGA and GEO (GSE58828, GSE53137),^[^ ^15^ ^]^ alongside ribosome‐associated RNAs extracted from GEO datasets (GSE79539, GSE111866, GSE129757).^[^ ^16^, ^17^ ^]^ Subsequently, lncRNAs exhibiting a fold‐change ≥2 were meticulously chosen for Venn analysis, culminating in the recognition of six promising candidate lncRNAs: DLGAP1‐AS2, HCP5, LINC00152, UCA1, MIR17HG, and H19, all demonstrating notable coding potential and significantly elevated expression levels in GC (**Figure** **1**A; Figure S1A, Supporting Information). To assess their translational capability, a combination of polysome profiling and quantitative reverse transcription real‐time polymerase chain reaction (RT‐qPCR) was employed. These experimental setups were designed to gauge the mRNA expression levels of non‐ribosomal entities, 40–80S ribosomal complexes and multibody components, as delineated previously.^[^ ^18^ ^]^ Outcome analysis revealed that amongst the cohort of six candidate lncRNAs, HCP5 emerged as the most enriched species within the polysome fraction of GC cells, cementing its exceptional potential for encoding proteins (Figure 1B; Figure S1B, Supporting Information). HCP5 was originally annotated as a ncRNA (NR_040662.1). Through analysis of the TCGA and GEO databases (GSE58828, GSE53137), we observed markedly elevated levels of lncRNA HCP5 expression in GC tissues as opposed to adjacent normal tissues (Figure S1C, Supporting Information). Its abnormal expression leads to malignant proliferation and metastasis of GC cells, inhibition of apoptosis and enhancement of chemotherapy resistance, suggesting that HCP5 has a carcinogenic effect and plays an important role in the occurrence of GC.^[^ ^19^, ^20^, ^21^ ^]^ Nonetheless, the mechanistic underpinnings of HCP5 primarily entail functioning as a microRNA sponge that regulates the expression of downstream target genes.^[^ ^22^, ^23^ ^]^ Further investigation is warranted to elucidate the coding potential of HCP5 in GC, as well as the biological functions and underlying mechanisms of its products.

In order to investigate the coding potential of HCP5, ORF Finder analysis predicted that HCP5 contained multiple ORFs with coding potential (Figure S1D, Supporting Information). To determine the translational potential of those ORFs, we generated various ORFs‐Flag constructs by fusing a Flag‐tag at the C‐terminus of each ORF. Subsequent detection of these Flag‐fusion proteins via western blotting revealed that only ORF15 was capable of translation, while the exogenous expression of ORF15‐Flag was identified by MS (Figure 1C; Figure S1E, Supporting Information). Notably, further in vitro translation experiments corroborated that the full‐length lncRNA HCP5 transcribed extracellularly could translate microproteins in the presence of ^35^S‐methionine, whereas mutations in the start codon of ORF15 impaired extracellular translation of lncRNA HCP5 (Figure 1D). The above preliminary identification indicates that the 399 nt ORF15 of lncRNA HCP5 is situated on exon 2 of chromosome 6, which may encode a 132aa microprotein named HCP5‐132aa (Figure 1E). To determine whether the start codon of HCP5‐132aa was truly active, we generated a series of ORF15‐GFP mut and ORF15‐Flag constructs by fusing a GFP mut‐tag (the in‐frame start codon was mutated from ATG into ATT) or Flag‐tag at the C‐terminus of ORF15 (Figure 1F). Substantial expression of HCP5‐132aa‐GFP and HCP5‐132aa‐Flag fusion proteins was observed in cells transfected with ORF15‐GFP mut and ORF15‐Flag constructs via microscope, western blotting and immunofluorescence staining, whereas the mutation of the HCP5‐132aa start codon (ATG to ATT) abolished the translation of the predicted ORF15 in HCP5 (Figure 1G–I). Collectively, these data indicate that HCP5 actually encodes a microprotein HCP5‐132aa.

To investigate the presence and expression of the natural, endogenous HCP5‐132aa in GC tissues and cells, we isolated proteins from GC tissues and cells. Subsequently, the unique amino acid sequence identified through MS analysis of the SDS‐PAGE gel ≈15 kDa provides strong evidence supporting the presence of natural endogenous HCP5‐132aa (Figure 1J,K). Furthermore, we generated an antibody targeting HCP5‐132aa and evaluated its expression in 70 pairs of matched fresh primary GC tissues and corresponding non‐tumor tissues via western blotting. The results demonstrated significantly elevated levels of HCP5‐132aa in primary GC tissues compared to their non‐tumor counterparts (Figure 1L). Meanwhile, the existence of natural endogenous HCP5‐132aa was confirmed in GC cells through western blotting and immunofluorescence staining (Figure 1M; Figure S1F, Supporting Information). In aggregate, these data suggest that HCP5‐132aa is endogenously, naturally produced in human GC tissues and cells.

### 2.2 HCP5‐132aa Promotes the Proliferation of GC Cells In Vitro and In Vivo

To investigate the functions of HCP5‐132aa in GC tumorigenesis, we employed CRISPR/Cas9 technology to construct stable knockout of HCP5‐132aa in MKN‐45 and NCI‐N87 cells, while stably overexpressed HCP5‐132aa in HGC‐27 cells (Figure S2A,B, Supporting Information). Furthermore, neither the knockout nor over‐expression of HCP5‐132aa affected the expression of lncRNA HCP5 (Figure S2C, Supporting Information). CCK‐8 assay, colony formation assay and Edu staining experiments demonstrated that HCP5‐132aa knockout significantly inhibited the proliferation and colony formation of GC cells, whereas its over‐expression exerted the opposite effect (Figure S2D–H, Supporting Information). These results preliminarily suggest that HCP5‐132aa promotes the malignant proliferation of GC.

**Figure 2:**
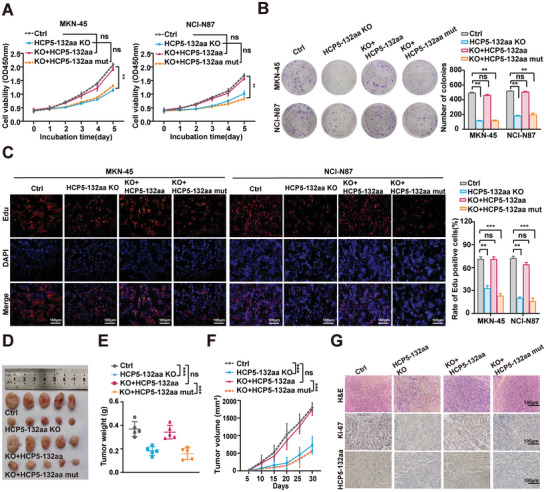
HCP5‐132aa, but not lncRNA HCP5, stimulates GC tumorigenesis in vitro and in vivo. A–C) Four groups of stable GC cell lines: Ctrl, HCP5‐132aa KO, KO+HCP5‐132aa, and KO+ HCP5‐132aa mut, were used for CCK‐8 (*n* = 5) (A), colony formation (*n* = 3) (B), and Edu staining (*n* = 3) (C) assay to detect the proliferative activity of GC cells. D) The xenograft mouse model was established using the aforementioned cells, and representative images of tumors from the xenograft model were displayed. E,F) Tumor volume and weight of subcutaneous xenografts in nude mice were measured and calculated every week (*n* = 5). G) Representative H&E staining of xenograft tissues and IHC staining of Ki‐67 and HCP5‐132aa are shown. scale bar: 100 µm. Data are represented as mean ± SD. Differences between the groups were evaluated using One‐way ANOVA with Tukey's Multiple Comparison test (A, B, C, E, F), ns indicates no significance, **p < 0.01, ***p < 0.001.

To further substantiate the role of HCP5‐132aa in promoting the malignant proliferation of GC cells, we generated several stable GC cell lines, including HCP5‐132aa KO, HCP5‐132aa KO with re‐expression of HCP5‐132aa (KO+ HCP5‐132aa), HCP5‐132aa KO with re‐expression of HCP5‐132aa mut (KO+ HCP5‐132aa mut), and a negative control (Ctrl). Then, the efficiency of HCP5‐132aa expression in these GC cell lines was assessed using western blotting (Figure S2I, Supporting Information). Subsequently, a series of loss‐ and gain‐ of function experiments were conducted to investigate the role of HCP5‐132aa in GC cells. The results revealed that alterations induced by HCP5‐132aa knockout were restored to control levels upon HCP5‐132aa re‐expression, whereas over‐expression of HCP5‐132aa mut (initiation codon mutation of ORF15) failed to rescue the observed cellular phenotype (**Figure** **2**A–C).

Meanwhile, we examined the role of HCP5‐132aa in GC development in vivo. A BALB/c nude mouse subcutaneous tumor model was constructed with Ctrl, HCP5‐132aa KO, KO+HCP5‐132aa, and KO+HCP5‐132aa mut GC cells. The xenografts derived from HCP5‐132aa KO cells exhibited significantly reduced tumor growth in vivo, which was restored upon reintroduction of HCP5‐132aa but not HCP5‐132aa mut (Figure 2D–F). In addition, H&E staining, immunohistochemistry, and western blotting were employed to detect the expression of HCP5‐132aa and Ki‐67 in tumor tissues from the various xenograft nude mice groups (Figure 2G; Figure S2J, Supporting Information). The results confirmed the successful establishment of xenograft nude mouse models and demonstrated that in vivo knockout of HCP5‐132aa impacted tumor proliferation, consistent with the aforementioned cellular phenotype conclusions. Collectively, the above findings underscore that HCP5‐132aa functions as an oncoprotein by promoting the proliferation of GC cells.

### 2.3 HCP5‐132aa Enhances the Interaction of RNA Binding Proteins YBX1 and ELAVL1

**Figure 3:**
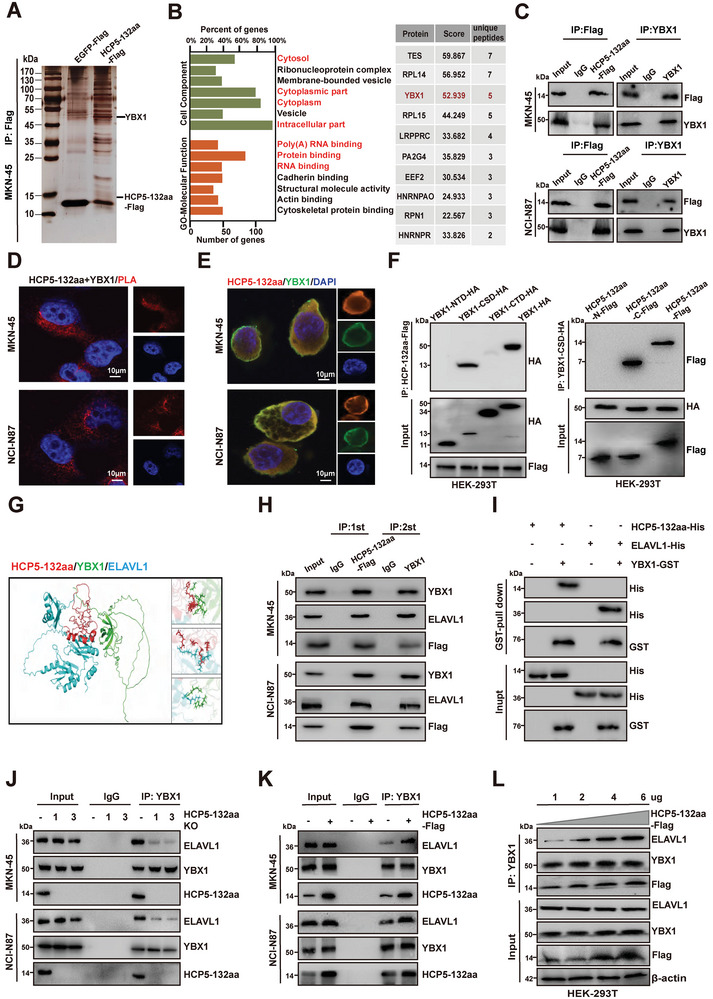
HCP5‐132aa enhances the binding of RNA binding proteins YBX1 and ELAVL1 in GC cells. A) Immunoprecipitation was performed using an anti‐Flag antibody in GC cells with stable expression of EGFP‐Flag or HCP5‐132aa‐Flag, followed by separation of the proteins via SDS‐PAGE and displayed with silver staining. B) GO functional enrichment analysis of the 152 proteins identified through MS analysis to bind with HCP5‐132aa (left), along with a representation of the top ten RNA‐binding proteins that bind with HCP5‐132aa, ranked by specific peptide sequences and MS scores (right). C,D) Co‐IP and PLA confirmed the interaction between HCP5‐132aa and YBX1 in GC cells, scale bar: 10 µm. E) Immunofluorescence staining revealed the co‐localization of HCP5‐132aa and YBX1 in GC cells, scale bar: 10 µm. F) HEK‐293T cells were transfected with Flag‐tagged HCP5‐132aa and HA‐tagged full‐length YBX1 or YBX1 fragments, and subjected to Co‐IP using an anti‐HA antibody (left). In addition, HEK‐293T cells were transfected with HA‐tagged YBX1‐CSD and Flag‐tagged HCP5‐132aa truncation mutants, followed by Co‐IP with an anti‐Flag antibody (right). G) AlphaFold software predicts the schematic representation of HCP5‐132aa/YBX1/ELAVL1 trimer. H) Sequential immunoprecipitation assays revealed the formation of a trimeric complex composed of HCP5‐132aa/YBX1/ELAVL1 in GC cell lines. I) In vitro pulldown assays were performed using bacterial purified HCP5‐132aa‐His, ELAVL1‐His, and YBX1‐GST to confirm the direct interaction between HCP5‐132aa/YBX1/ELAVL1. J,K) Co‐IP was employed to examine the interaction between YBX1 and ELAVL1 in HCP5‐132aa stable knockout or overexpressing GC cell lines. L) YBX1 and ELAVL1 co‐transfected into HEK‐293T cells with HCP5‐132aa‐Flag in a dose‐dependent manner.

HCP5‐132aa is an uncharacterized microprotein lacking known domains or motifs and exhibiting no homology with other proteins. To further investigate the mechanisms by which HCP5‐132aa acts in GC progression, immunoprecipitation was carried out in GC cells with stable HCP5‐132aa expression, followed by silver staining to visualize interacting proteins (**Figure** **3**A). Compared to the control group with stable expression of EGFP‐Flag, 152 candidate proteins that interacted with HCP5‐132aa were identified by subsequent MS analysis in the stable HCP5‐132aa expression group. Gene Ontology (GO) analyses revealed that the majority of proteins interacting with HCP5‐132aa were located in the cytoplasm, primarily comprised of protein‐binding, RNA‐binding and poly(A) binding proteins, suggesting that HCP5‐132aa may interact with RNA‐binding proteins to promote cancer development (Figure 3B, left). Subsequently, we ranked the RNA‐binding proteins interacting with HCP5‐132aa based on MS scores and the number of specific peptides. Among the top ten candidate proteins, Y‐box binding protein 1 (YBX1) particularly attracted our attention (Figure 3B; right and Table S5, Supporting Information). Previous reports have demonstrated that high expression of YBX1 was associated with the progression and poor prognosis of various cancers, including GC, lung cancer, breast cancer, and colorectal cancer.^[^ ^24^, ^25^ ^]^ Moreover, YBX1 regulates malignant phenotypes of cancer cells by participating in physiological processes such as mRNA splicing, translation regulation, stability maintenance, and DNA damage repair.^[^ ^26^, ^27^ ^]^ Interestingly, unique peptide fragments of YBX1 co‐immunoprecipitated by HCP5‐132aa were identified in the MS data, hinting at the potential interaction between YBX1 and HCP5‐132aa in GC cells (Figure S3A, Supporting Information). Therefore, we have ultimately chosen YBX1 as the target protein of HCP5‐132aa for further investigation.

As expected, co‐immunoprecipitation (Co‐IP) assay validated the interaction between HCP5‐132aa and YBX1 in GC cells, while the proximity ligation assay (PLA) further corroborated this interaction (Figure 3C,D). Additionally, immunofluorescence staining demonstrated the co‐localization of HCP5‐132aa and YBX1 in the cytoplasm of GC cells (Figure 3E). YBX1 consists of three domains: the alanine/proline‐rich N‐terminal domain (A/P domain), the cold shock domain (CSD) and the large C‐terminal domain (CTD) with alternating clusters of positively and negatively charged amino acid residues.^[^ ^28^ ^]^ To further determine the specificity of the interaction, YBX1 truncated constructs with a C‐terminal HA‐tag were co‐expressed with HCP5‐132aa‐Flag in HEK‐293T cells (Figure S3E, Supporting Information). Subsequent Co‐IP experiments combined with western blotting demonstrated that only constructs containing the CSD of YBX1 retained the ability to interact with HCP5‐132aa (Figure 3F). Meanwhile, by reciprocal Co‐IP analysis with flag‐tagged truncation mutants of HCP5‐132aa, we also identified that only the C‐terminal region (67‐132aa) of HCP5‐132aa, rather than the N‐terminal region (1‐66aa), was able to interact with the CSD domain of YBX1 (Figure 3F).

Next, we investigated whether HCP5‐132aa affects the RNA‐binding protein function of YBX1 during GC progression. We observed that HCP5‐132aa knockout or YBX1 knockdown did not impact the expression of each other's RNA and protein, respectively (Figure S3B–D, Supporting Information). A previous study demonstrated that YBX1, as an RNA binding protein, maintains the stability of target mRNA at the post‐transcriptional level by recruiting ELAVL1, thereby modulating the outcome of cellular programs such as myogenesis.^[^ ^29^ ^]^ Surprisingly, the presence of ELAVL1 was also detected among the proteins interacting with HCP5‐132aa identified by MS (Figure S3F, Supporting Information). Furthermore, the interaction between HCP5‐132aa and ELAVL1 was confirmed using Co‐IP, PLA and immunofluorescence staining, showing predominant co‐localization in the cytoplasm (Figure S3G–I, Supporting Information). Therefore, we hypothesized that HCP5‐132aa might be involved in the formation of the YBX1/ELAVL1 complex. In order to verify this conjecture, we initially predicted using AlphaFold software that HCP5‐132aa/YBX1/ELAVL1 could potentially form trimers (Figure 3G). Sequential immunoprecipitation assays confirmed that HCP5‐132aa formed trimer complexes with YBX1 and ELAVL1, and in vitro GST pulldown assay confirmed that HCP5‐132aa could directly promote the interaction between YBX1 and ELAVL1, indicating that HCP5‐132aa/YBX1/ELAVL1 formed a trimer complex (Figure 3H,I). Additionally, by constructing a series of truncated forms of ELAVL1, Co‐IP demonstrated that the RRM2 domain of ELAVL1 interacted with the N‐terminal of HCP5‐132aa (Figure S3J, Supporting Information). Given YBX1's predominant interaction with the C‐terminal of HCP5‐132aa, it is proposed that HCP5‐132aa acts as a bridging molecule facilitating the interaction between YBX1 and ELAVL1. PLA experiments validated this hypothesis, showing that HCP5‐132aa knockout significantly attenuated the binding between YBX1 and ELAVL1 (Figure S3K, Supporting Information). Additionally, Co‐IP experiments indicated that knockout of HCP5‐132aa in GC cells diminished the interaction between YBX1 and ELAVL1, while ectopic expression of HCP5‐132aa strengthened their interaction in a dose‐dependent manner (Figure 3J–L). Collectively, these results indicate that HCP5‐132aa acts as a bridge protein to enhance YBX1 binding to ELAVL1 in GC cells.

### 2.4 HCP5‐132aa/YBX1/ELAVL1 Regulates the mRNA Stability of SLC7A11 and G6PD

**Figure 4:**
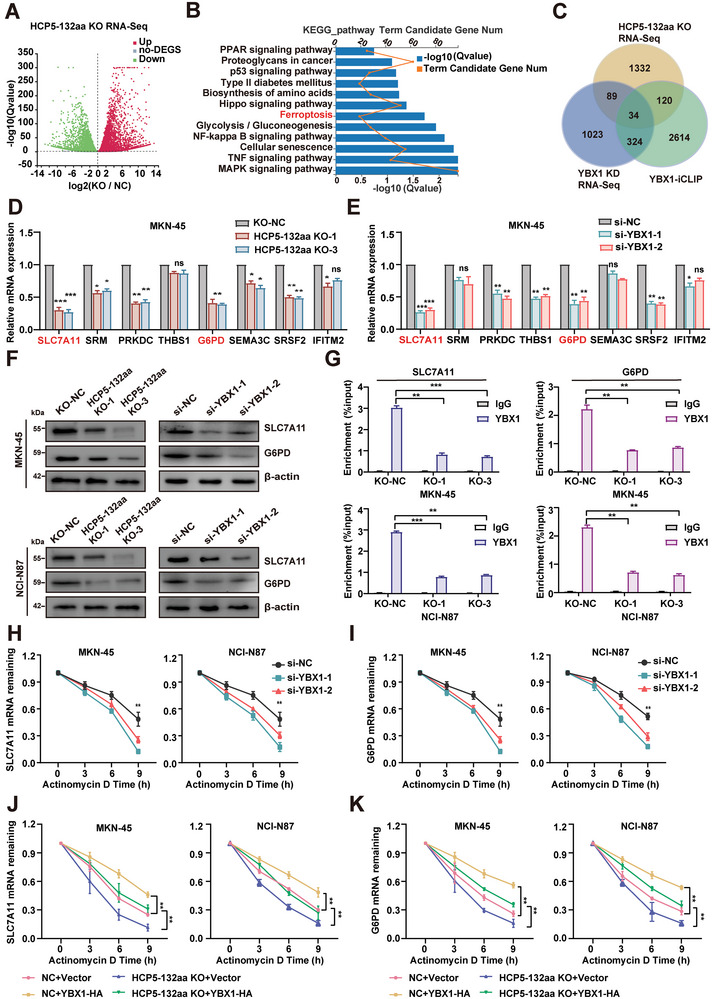
HCP5‐132aa mediates YBX1 regulation of SLC7A11 and G6PD mRNA stability. A) The volcano plot displays the outcomes of RNA‐seq analysis for genes that exhibit differential expression between KO‐NC and HCP5‐132aa KO GC cells. B) The KEGG analyses identified signaling pathways that were significantly impacted as a result of HCP5‐132aa knockout in GC cells. C) The Venn diagram shows that the RNA‐seq results of HCP5‐132aa knockout and YBX1 knockdown were overlapped with the iCLIP data of YBX1 (http://www.sibcb.ac.cn/hui.asp) to identify HCP5‐132aa / YBX1 target genes. D,E) RT‐qPCR was performed to evaluate the impact of YBX1 knockdown or HCP5‐132aa knockout on the mRNA expression of eight candidate target genes in GC cells (*n* = 3). F) Western blotting analysis to evaluate the protein levels of SLC7A11 and G6PD in GC cells with stable HCP5‐132aa knockout or YBX1 knockdown. G) YBX1 antibodies were applied for RIP assay in HCP5‐132aa knockout GC cells, followed by RT‐qPCR quantification to assess the binding of YBX1 with SLC7A11 and G6PD mRNA (*n* = 3). H,I) Following treatment with ActD, RT‐qPCR analysis was conducted to determine the mRNA half‐life of SLC7A11 and G6PD in GC cells in which YBX1 were knocked down (*n* = 3). J,K) The increase in SLC7A11 and G6PD mRNA half‐life caused by YBX1 expression was inhibited by the knockout of HCP5‐132aa (*n* = 3). Data are represented as mean ± SD. Differences between the groups were evaluated using Student's t‐test (D, E, G, H, I) or One‐way ANOVA with Tukey's Multiple Comparison test (J, K), ns indicates no significance, *p < 0.05, **p < 0.01, ***p < 0.001.

Subsequently, we investigated whether HCP5‐132aa, through the RNA binding protein function of YBX1, modulated the fate and function of its downstream target mRNA at the post‐transcriptional level and thereby promoted the malignant phenotype of GC cells. First, to characterize the downstream target of HCP5‐132aa, we performed RNA‐seq analysis and identified 837 up‐regulated genes and 812 down‐regulated genes in HCP5‐132aa knockout GC cells (**Figure** **4**A). Further analysis using the KEGG pathway revealed that these differentially expressed genes were concentrated in signaling pathways associated with abnormal tumor proliferation and progression, such as the MAPK cascade, NF kappa B signaling pathway and ferroptosis, etc (Figure 4B). This analysis also verified the above functional results indicating that HCP5‐132aa regulates the malignant proliferation of GC cells. Subsequently, we used siRNA knockdown of YBX1 in GC cells and performed RNA‐Seq analysis to identify downstream target genes co‐regulated by HCP5‐132aa and YBX1. We analyzed the RNA‐Seq data under conditions of HCP5‐132aa knockout and YBX1 knockdown, as well as YBX1‐iCLIP data that specifically captured RNA transcripts bound to YBX1.^[^ ^30^ ^]^ The comprehensive analysis revealed that HCP5‐132aa and YBX1 together regulated a total of 34 target genes (Figure 4C). Then, we performed RT‐qPCR to measure the expression levels of the top eight differentially expressed candidate genes and found that SLC7A11 was the most significantly down‐regulated gene in GC cells with either HCP5‐132aa knockout or YBX1 knockdown (Figure 4D,E; Figure S4A,B, Supporting Information). SLC7A11, a component of system Xc−, has recently been reported to be upregulated in several human cancers, including GC, contributing to various biological processes that facilitate tumor progression, partly by inhibiting ferroptosis.^[^ ^31^ ^]^ Furthermore, we observed a significant downregulation of G6PD, another ferroptosis‐associated gene, in GC cells following HCP5‐132aa knockout or YBX1 knockdown. Subsequently, we employed western blotting to measure the significantly down‐regulated SLC7A11 and G6PD protein expression in GC cells with HCP5‐132aa knockout or YBX1 knockdown (Figure 4F). Therefore, we selected SLC7A11 and G6PD as downstream target genes of HCP5‐132aa /YBX1 for further investigation.

Previous studies have confirmed that HCP5‐132aa acts as an accessory protein that enhances the assembly of the YBX1/ELAVL1 complex. As expected, knockdown of ELAVL1 significantly decreased the mRNA and protein expression levels of SLC7A11 and G6PD, similar to the effects observed upon knocking out HCP5‐132aa or knocking down YBX1 (Figure S4C,D, Supporting Information). Given that YBX1 enhances the stability of target RNA by recruiting ELAVL1,^[^ ^29^ ^]^ it is conceivable that HCP5‐132aa may modulate the stability of SLC7A11 and G6PD mRNA through the RNA‐binding protein function of YBX1/ELAVL1. The RNA‐binding protein immunoprecipitation assay revealed that YBX1 and ELAVL1 demonstrated an ability to bind with SLC7A11 or G6PD mRNA in GC cells; however, their binding capacity was noticeably impaired following the knockout of HCP5‐132aa (Figure 4G; Figure S4E,F, Supporting Information). We then performed RNA decay assays using the transcription inhibitor actinomycin D at the indicated time. As shown in the curves, YBX1 or ELAVL1 knockdown significantly reduced the stability of SLC7A11 and G6PD mRNA (Figure 4H,I; Figure S4G,H, Supporting Information). Conversely, up‐regulation of YBX1 or ELAVL1 led to an improvement in the stability of SLC7A11 and G6PD mRNA, but this phenomenon was attenuated after the knockout of HCP5‐132aa (Figure 4J,K; Figure S4I,J, Supporting Information). These results suggest that the HCP5‐132aa/YBX1/ELAVL1 complex affects the mRNA stability of SLC7A11 and G6PD at the post‐transcriptional level in GC cells.

### 2.5 HCP5‐132aa Facilitates YBX1 Recognition of m^5^C Modifications in SLC7A11 and G6PD mRNA

**Figure 5:**
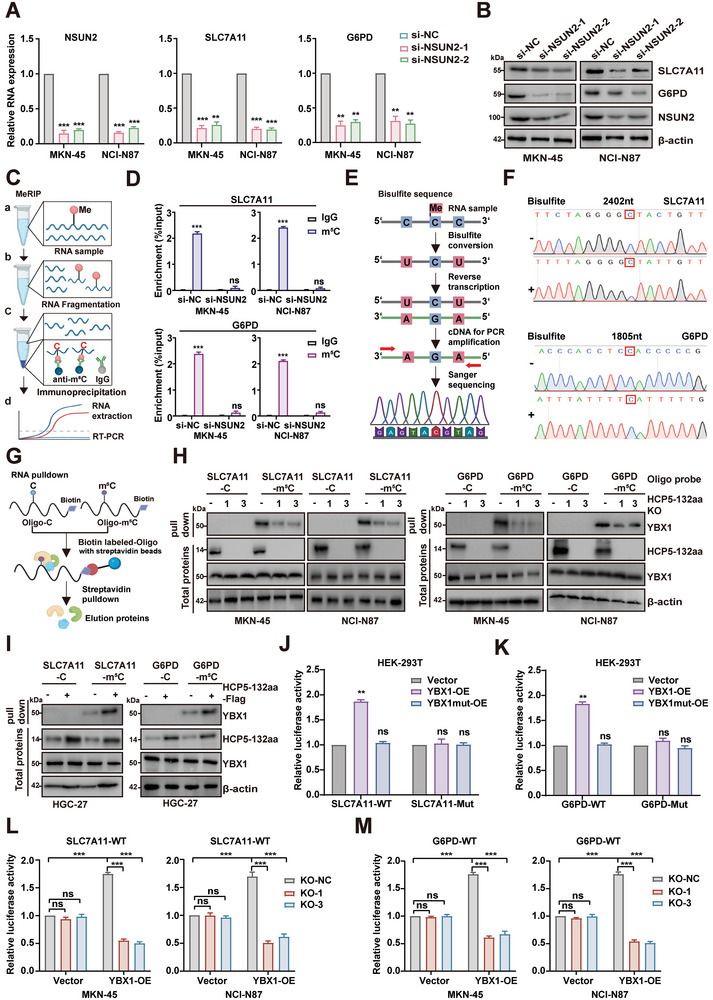
HCP5‐132aa facilitates YBX1 recognition of m^5^C modifications in SLC7A11 and G6PD mRNA. A,B) The mRNA and protein expression levels of NSUN2, SLC7A11 and G6PD were evaluated by RT‐qPCR and western blotting in NSUN2‐depleted GC cells (*n* = 3). C) Schematic diagram of MeRIP experiment using m^5^C antibody. D) MeRIP followed by RT‐qPCR analysis was used to assess the m^5^C level of SLC7A11 and G6PD mRNA in GC cells with NSUN2 knockdown (*n* = 3). E) Schematic diagram of Bisulfite‐PCR pyrosequencing experiment. F) PCR amplification was performed on cDNA obtained from bisulfite‐treated RNA, followed by Sanger sequencing to verify the m^5^C site in SLC7A11 and G6PD mRNA. G) Schematic diagram of RNA pull‐down experiment. H,I) RNA pull‐down assays were conducted to investigate the in vitro binding of YBX1 to unmethylated or m^5^C‐methylated oligonucleotides of SLC7A11 or G6PD mRNA in GC cells with either stable knockout or overexpression of HCP5‐132aa. J,K) The relative luciferase activity of SLC7A11‐WT or SLC7A11‐Mut (G6PD‐WT or G6PD‐Mut) was measured in HEK‐293T cells co‐transfected with YBX1‐OE, YBX1 mut‐OE, or control, respectively (*n* = 3). L,M) Relative luciferase activity of SLC7A11‐WT or G6PD‐WT in HCP5‐132aa KO or control HEK‐293T cells with expression of YBX1 (*n* = 3). Data are represented as mean ± SD. Differences between the groups were evaluated using Student's t‐test (A, D, J, K) or One‐way ANOVA with Tukey's Multiple Comparison test (L, M), ns indicates no significance, **p < 0.01, ***p < 0.001.

Recent discoveries have demonstrated that aberrations in epigenetic regulation, such as RNA methylation, are crucial hallmarks of tumor initiation, progression and recurrence.^[^ ^32^, ^33^ ^]^ 5‐Methylcytosine (m^5^C) is one of the most well‐known and conserved RNA modifications occurring in various eukaryotic RNA types, including rRNA, lncRNA, tRNA, and mRNA.^[^ ^34^, ^35^, ^36^, ^37^ ^]^ It is known that YBX1 is an m^5^C reader protein that recognizes m^5^C‐modified mRNAs and enhances their stability by recruiting ELAVL1.^[^ ^38^ ^]^ Based on this knowledge, we hypothesized that the mRNAs of SLC7A11 and G6PD might be modified by m^5^C methylation. Since NOP2/Sun RNA methyltransferase family member 2 (NSUN2) is a major enzyme responsible for m^5^C modification in mRNA in mammals,^[^ ^39^ ^]^ our initial examination centered on determining whether NSUN2 played a role in the m^5^C modification of SLC7A11 and G6PD mRNA. In subsequent experiments, we observed a significant decrease in the expression of SLC7A11 and G6PD at both the mRNA and protein levels after silencing NSUN2 in GC cells (**Figure** **5**A,B). MeRIP analysis using anti‐m^5^C antibodies confirmed the m^5^C modification in SLC7A11 and G6PD mRNA, which decreased when NSUN2 was depleted (Figure 5C,D). Furthermore, bisulfite‐PCR sequencing (BPS) revealed that the m^5^C methylation modification sites in SLC7A11 and G6PD mRNAs were C2402 and C1805 nt, respectively, both of which are located in the 3′UTR region (Figure 5E,F). Taken together, these findings indicate that SLC7A11 and G6PD mRNAs bear m^5^C modifications.

It is known that RNA molecules undergoing methylation modification require reader proteins to recognize their methylation sites, which in turn regulate various biological processes such as RNA stability, RNA export, RNA translation, etc.^[^ ^40^, ^41^, ^42^, ^43^ ^]^ Given that YBX1 is an m^5^C reader protein involved in regulating the mRNA stability of SLC7A11 and G6PD, it is reasonable to assume that YBX1 recognizes the m^5^C modifications in their mRNA molecules. To test this hypothesis, methylated single‐stranded RNA oligonucleotides targeting the m^5^C modification sites of SLC7A11 and G6PD mRNA (SLC7A11‐m^5^C‐oligo and G6PD‐m^5^C‐oligo) were synthesized and used in RNA pull‐down experiments in GC cells. Non‐methylated single‐stranded RNAs with shared sequences (SLC7A11‐C‐oligo and G6PD‐C‐oligo) were employed as controls (Figure 5G). The results indicated that YBX1 selectively binds to the methylated single‐stranded RNA oligonucleotides but not to the unmethylated ones, supporting the idea that YBX1 can recognize the m^5^C modifications in SLC7A11 and G6PD mRNAs (Figure 5H). Furthermore, we found that knockout of HCP5‐132aa significantly weakened the binding of YBX1 to m^5^C‐RNA oligonucleotides in GC cells, while its overexpression enhanced this binding (Figure 5I). We further constructed luciferase reporter systems (pMIR‐SLC7A11‐WT, pMIR‐G6PD‐WT) containing a 3′‐UTR enriched in m^5^C modifications, as well as mutated m^5^C sites (pMIR‐SLC7A11‐mut, pMIR‐G6PD‐mut), and co‐transfected them with YBX1 or YBX1mut (having a mutated m^5^C recognition domain) into HEK‐293T cells. The results showed that the expression of YBX1 induced luciferase activity, which was significantly inhibited by the mutated m^5^C sites. Overexpression of YBX1mut, which cannot recognize m^5^C‐modified RNA, completely abolished the luciferase activity (Figure 5J,K). Additionally, knocking out HCP5‐132aa in GC cells resulted in a significant decrease in luciferase activity induced by YBX1 compared to the control group (Figure 5L,M). These results suggest that HCP5‐132aa regulates the recognition of m^5^C modifications in SLC7A11 and G6PD mRNA by YBX1, thereby enhancing their stability.

### 2.6 HCP5‐132aa Suppresses Ferroptosis and Promotes the Proliferation of GC Cells via SLC7A11 and G6PD

Ferroptosis is a novel form of programmed cell death whose dysfunction has been associated with various diseases, especially cancer.^[^ ^44^ ^]^ The main characteristics of ferroptosis are lipid peroxidation and accumulation of ferrous ions. As a key regulatory protein in ferroptosis, SLC7A11 regulates the biosynthesis of reduced glutathione (GSH) and eliminates intracellular lipid reactive oxygen species (lipid ROS) to prevent cell death induced by membrane lipid peroxidation.^[^ ^45^ ^]^ Additionally, G6PD plays an important role in the pentose phosphate pathway as it controls the generation of intracellular reduced equivalent NADPH, which leads to the conversion of oxidized glutathione (GSSG) to reduced GSH and prevents cells from undergoing ferroptosis due to lipid peroxidation.^[^ ^46^ ^]^ Interestingly, the KEGG enrichment analysis of the HCP5‐132aa KO RNA‐seq data also indicated an enrichment of genes regulated by HCP5‐132aa in the ferroptosis pathway. Next, we aimed to determine whether HCP5‐132aa inhibits ferroptosis in GC cells by regulating SLC7A11 and G6PD.

**Figure 6:**
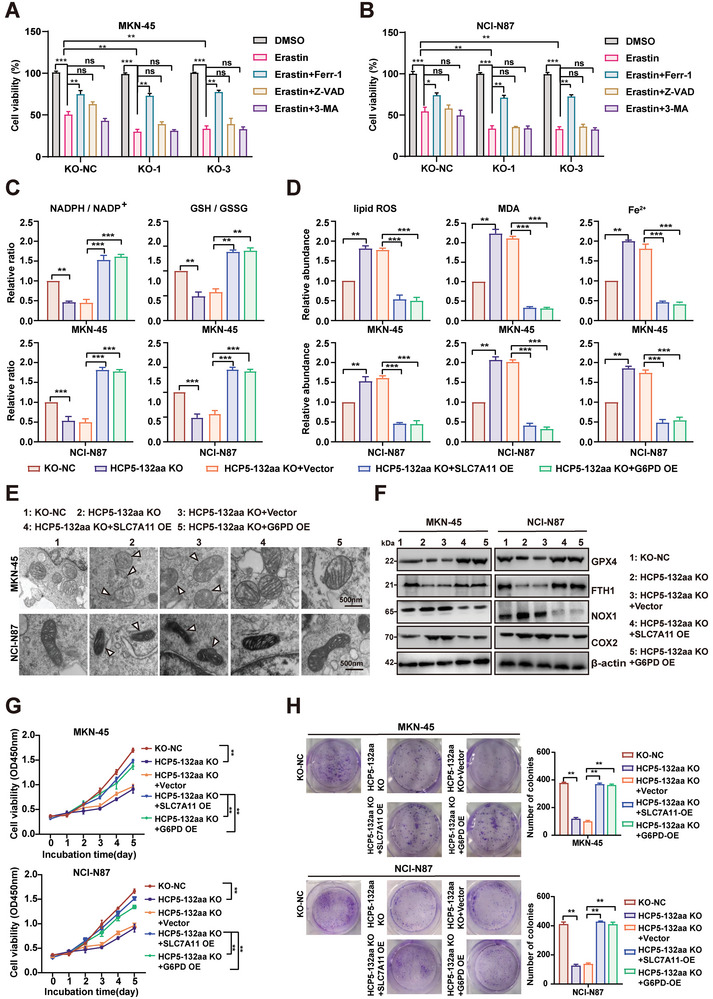
HCP5‐132aa suppresses ferroptosis and promotes the proliferation of GC cells via SLC7A11 and G6PD. A,B) The cell viability of HCP5‐132aa knockout GC cell lines was assessed by CCK‐8 assays after treatment with Erastin (10 µm), Erastin and Ferr‐1 (2 µm), Erastin and Z‐VAD (10 µm), or Erastin and 3‐MA, respectively (*n* = 5). C,D) The overexpression of SLC7A11 and G6PD was separately performed in HCP5‐132aa knockout GC cells, and empty vector was used as a control. The NADPH/NADP and GSH/GSSG ratios, MDA and ferrous ion levels in GC cells were measured using Coenzyme II NADP(H) Assay, GSH and MDA Assay kits, and Iron Detection Reagent kit, respectively. The cellular ROS level was determined using C11‐BODIPY fluorescent probe (*n* = 3). E) Transmission electron microscopy was performed to evaluate the change of mitochondrial ultrastructure in the above indicated GC cells after treated with Erastin (10 µm), scale bar, 500 nm. F) The expression levels of iron‐regulatory proteins, including GPX4, FTH1, NOX1, and COX2, were analyzed by western blotting in the above indicated GC cells. G,H) SLC7A11 and G6PD were individually overexpressed in GC cells with HCP5‐132aa knockout, and CCK‐8 (*n* = 5) and colony formation assays (*n* = 3) were performed to evaluate the proliferative activity of the cells. Data are represented as mean ± SD. Differences between the groups were evaluated using One‐way ANOVA with Tukey's Multiple Comparison test (A, B, C, D, G, H), ns indicates no significance, *p < 0.05, **p < 0.01, ***p < 0.001.

Initially, we used erastin to induce ferroptosis in control and HCP5‐132aa knockout cells, as evidenced by cellular viability assays showing that the absence of HCP5‐132aa significantly promoted erastin‐induced ferroptosis. Then, GC cells were treated with different types of cell death inhibitors. The cellular viability assays showed that knockdown of HCP5‐132aa sensitized GC cells to erastin‐induced growth inhibition over time, which could be rescued by the ferroptosis inhibitor Ferrostatin‐1 (Ferr‐1) but not by the apoptosis inhibitor Z‐VAD‐FMK or the autophagy inhibitor 3‐MA (**Figure** **6**A,B). Moreover, we proceeded to examine biochemical markers associated with ferroptosis. The knockout of HCP5‐132aa notably lowered the NADPH/NADP+ and GSH/GSSG ratios, while increasing lipid reactive oxygen species, the lipid peroxidation product MDA, and divalent iron ion content in GC cells (Figure S5A–C, Supporting Information). In parallel, western blotting analysis revealed downregulation of the ferroptosis‐associated proteins GPX4 and FTH1, along with upregulation of NOX1 and COX2 in HCP5‐132aa knockout GC cells (Figure S5D, Supporting Information). However, the induction of ferroptosis in GC cells caused by HCP5‐132aa knockout was disrupted upon re‐expression of HCP5‐132aa, while overexpression of HCP5‐132aa mut did not affect ferroptosis in GC cells. Overall, these results strongly indicate that HCP5‐132aa knockout promotes ferroptosis in GC cells. Ultimately, to further investigate whether HCP5‐132aa regulates ferroptosis in GC cells through SLC7A11 and G6PD, we re‐expressed SLC7A11 and G6PD in GC cells lacking HCP5‐132aa (Figure S6A, Supporting Information). The rescue experiment demonstrated that the aforementioned ferroptosis detection indicators could be restored upon re‐expression of SLC7A11 or G6PD in HCP5‐132aa knockout GC cells (Figure 6C–F). Additionally, observations from transmission electron microscopy (TEM) indicated that the severely shriveled mitochondria observed in GC cells with HCP5‐132aa knockout were reversed upon the re‐expression of SLC7A11 or G6PD (Figure 6E; Figure S5E, Supporting Information). These results indicate that HCP5‐132aa primarily inhibits ferroptosis in GC cells through SLC7A11 and G6PD.

To investigate the inhibitory effects of HCP5‐132aa on ferroptosis through SLC7A11 and G6PD, and how this promotes malignant proliferation in GC cells, we conducted an experiment where SLC7A11 and G6PD were re‐expressed in stable HCP5‐132aa knockout GC cells. The results clearly indicated that the re‐expression of SLC7A11 and G6PD reversed the inhibitory effects on cell proliferation caused by HCP5‐132aa knockout (Figure 6G,H). The high expression of SLC7A11 and G6PD in various malignant tumors has been closely associated with tumor growth and poor prognosis.^[^ ^47^, ^48^ ^]^ In this study, the expression level of SLC7A11 in 70 pairs of GC tissue specimens was detected by western blotting. The results showed that the expression of SLC7A11 in primary GC tissues was significantly higher than that in paired adjacent normal tissues (accounting for 80% of the total sample size) (Figure S6B, Supporting Information). The HPA database also showed that G6PD was significantly over‐expressed in GC tissues (Figure S6C, Supporting Information). Kaplan‐Meier survival analysis further showed that GC patients with lower expression levels of SLC7A11 or G6PD had higher overall survival rates (Figure S6D, Supporting Information). Moreover, the expression levels of SLC7A11 and G6PD were found to be significantly correlated with the expression level of HCP5‐132aa protein (Figure S6E, Supporting Information). Based on these data, it can be concluded that HCP5‐132aa primarily inhibits ferroptosis by regulating the expression of SLC7A11 and G6PD, thereby promoting malignant proliferation and tumor progression in GC.

### 2.7 HCP5‐132aa Upregulation Predicts Poor Prognosis and Offers a Promising Therapeutic Target in GC

**Figure 7:**
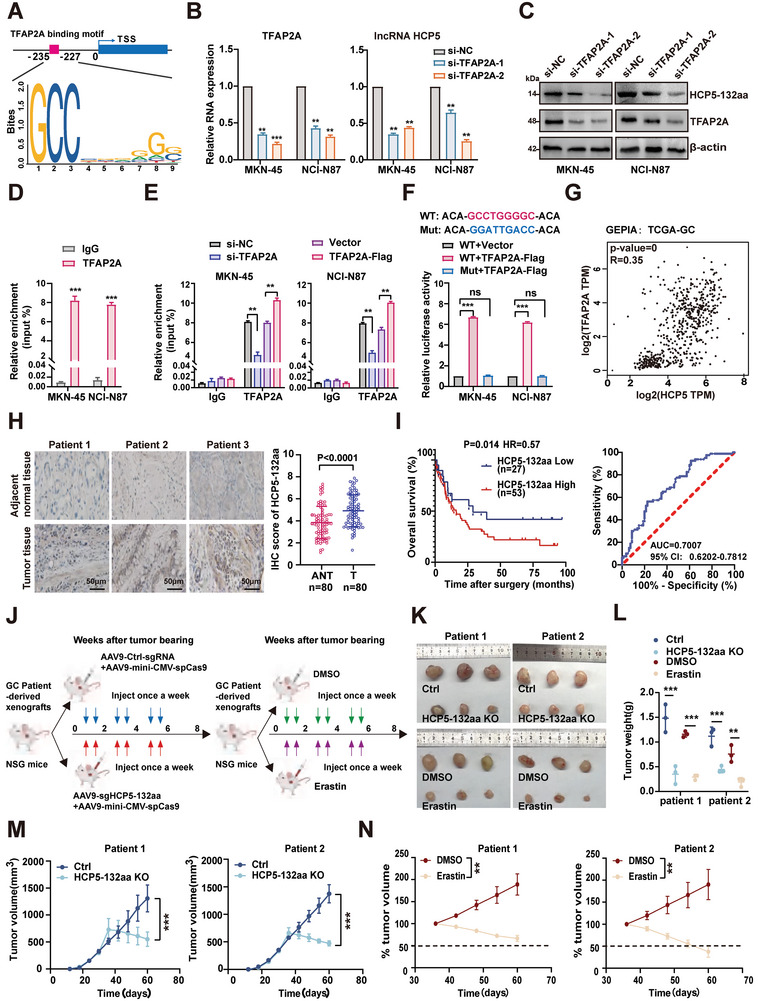
HCP5‐132aa upregulation predicts poor prognosis and offers a promising therapeutic target in GC. A) Sequence prediction of the promoter region of TFAP2A‐bound HCP5 by JASPAR. B,C) The mRNA and protein expression levels of TFAP2A and lncRNA HCP5 were evaluated by RT‐qPCR and western blotting in TFAP2A‐depleted GC cells (*n* = 3). D) The ChIP‐qPCR analysis confirmed the enrichment of TFAP2A and HCP5 promoter sequences in GC cells (*n* = 3). E) The enrichment of TFAP2A and KRT16 promoter sequences in TFAP2A silenced or overexpressed GC cells was verified by ChIP‐qPCR assay (*n* = 3). F) After co‐transfection of luciferase reporter plasmids containing or lacking the HCP5 core promoter sequence with TFAP2A into GC cells, the relative intensity of luciferase was measured (*n* = 3). G) The GEPIA analysis of TCGA‐GC data revealed a positive correlation between the expression levels of TFAP2A and HCP5 (Pearson R = 0.35, *p* < 0.05). H) Representative IHC images of HCP5‐132aa expression in GC tissues and adjacent paracancerous tissues were obtained, and their IHC scores were compared using a paired t‐test. I) The Kaplan‐Meier survival analysis demonstrated a positive correlation between high expression of HCP5‐132aa in GC and shorter overall survival of patients (left). ROC curve revealed that HCP5‐132aa is a valuable diagnostic biomarker for GC (right). J) The figure depicts a strategy involving CRISPR/Cas9‐mediated knockout of HCP5‐132aa in two GC patient‐derived xenograft (PDX) models (left). Following tumor engraftment, mice were intratumorally injected with AAV9‐sgHCP5‐132aa and AAV9‐mini‐CMV‐sgCas9 viruses (red arrows) at a 1:2 ratio, or with AAV9‐Ctrl‐sgRNA and AAV9‐mini‐CMV‐sgCas9 viruses (blue arrows), for 6 weeks. Concurrently, the impact of ferroptosis on tumor growth was assessed using these two GC‐PDX models (right), with mice receiving intraperitoneal injections of DMSO or erastin (30 mg k^−1^g i.v., weekly) for 6 weeks. K) The tumors generated in vivo by various GC‐PDX models are shown. L) The figure depicts the statistically analyzed tumor weights of each group of GC‐PDX models (*n* = 3). M, N) Each group of GC‐PDX tumor volumes was measured regularly, followed by statistical analysis at 60 days to assess the changes in tumor size for each PDX group (*n* = 3). Data are represented as mean ± SD. Differences between the groups were evaluated using Student's t‐test (B, D, M, N) or One‐way ANOVA with Tukey's Multiple Comparison test (E, F, L), ns indicates no significance, **p < 0.01; ***p < 0.001.

In this study, we observed that HCP5‐132aa was upregulated in GC and acted as an oncoprotein, promoting malignant proliferation of GC cells. Nevertheless, the mechanism responsible for the anomalous expression of HCP5‐132aa remained unclear. Transcriptional regulation is one of the indispensable components of the gene expression control system and directly governs gene expression.^[^ ^49^ ^]^ Herein, to investigate the potential mechanism underlying the elevated expression of HCP5‐132aa in GC, we utilized multiple transcription‐related databases to predict potential transcription factors (TFs) that might bind the promoter region of HCP5 in GC, identifying three TFs likely involved in regulating HCP5 transcription (Figure S7A, Supporting Information). Notably, TFAP2A exhibited the highest binding score (**Figure** **7**A; Figure S7B, Supporting Information). To investigate the correlation between TFAP2A and HCP5, we initially silenced TFAP2A in GC cells and observed significant reductions in HCP5 RNA expression and HCP5‐132aa protein levels (Figure 7B,C). Importantly, ChIP‐qPCR results revealed that HCP5 promoter DNA fragments were enriched by TFAP2A antibody pulldown, and that the efficiency of TFAP2A binding to the HCP5 promoter region decreased after TFAP2A silencing, but increased after TFAP2A overexpression (Figure 7D,E). After that, a dual‐luciferase reporter system with and without the HCP5 core promoter region was constructed, showing that the wild‐type site rather than the mutant site triggered the luciferase activity in TFAP2A‐overexpressed GC cells (Figure 7F). TFAP2A is known to be markedly overexpressed in GC,^[^ ^50^ ^]^ and consistent with this, GEPIA analysis confirmed the high expression of TFAP2A in GC (Figure S7C, Supporting Information). Additionally, Kaplan‐Meier analysis revealed that patients exhibiting elevated TFAP2A expression levels had inferior prognostic outcomes (Figure S7D, Supporting Information). Furthermore, correlation analysis indicated a positive correlation between the expression of TFAP2A and HCP5 (SLC7A11/G6PD) in GC (Figure 7G; Figure S7E,F, Supporting Information). Based on these findings, we postulate that TFAP2A plays a crucial role as a transcription factor for HCP5 and is involved in its upregulation in GC.

Subsequently, we used immunohistochemical methods to detect HCP5‐132aa expression in 80 pairs of GC and adjacent tissues and associated these with follow‐up survival data to investigate the clinical relevance of the aberrantly high expression of HCP5‐132aa in GC. The results showed that HCP5‐132aa was significantly upregulated in GC tissues compared to adjacent ones (accounting for 80% of the total samples), and its elevated expression was positively associated with larger tumor size, lymph nodes metastasis and advanced clinical stage (Figure 7H; Figure S8A and Table S1, Supporting Information). Kaplan‐Meier analysis showed that patients with higher expression levels of HCP5‐132aa in tumor had a poor prognosis, and we also constructed a receiver operating characteristic curve and calculated the area under the curve (AUC) with an AUC value of 0.86 for HCP5‐132aa (Figure 7I). These results indicate that HCP5‐132aa has the potential as a biological indicator for the diagnosis and prognosis of GC.

**Figure 8:**
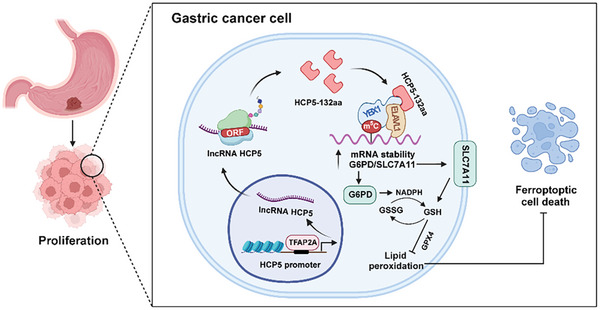
The working model illustrating HCP5‐132aa driving GC progression. Using bioinformatics analysis and polysome profiling assay, we identified and validated the coding potential of aberrantly highly expressed lncRNA HCP5 in GC, with its upregulation primarily mediated by the transcription factor TFAP2A binding to its promoter region, thereby enhancing its expression. The microprotein HCP5‐132aa, encoded by lncRNA HCP5, exhibits oncogenic protein functions both in vitro and in vivo. HCP5‐132aa enhances the formation of the RNA‐binding protein complex YBX1/ELAVL1, thereby facilitating YBX1 recognition of m^5^C modifications on SLC7A11 and G6PD mRNA, regulating their mRNA stability, and consequently inhibiting ferroptosis to promote malignant proliferation and tumor progression in GC.

In recent years, the CRISPR/Cas9 genome editing technology has been increasingly employed for disrupting genomic DNA in cancer cells and animal models, and has emerged as a valuable tool for exploring cancer therapies due to its remarkable efficiency and accuracy, so it holds great potential for therapeutic applications.^[^ ^51^ ^]^ In order to assess the therapeutic viability of targeting HCP5‐132aa in GC, we packaged adeno‐associated viruses (AAV) containing the CRISPR/Cas9 system ex vivo, followed by in vivo direct injection to specifically knock out HCP5‐132aa in patient‐derived xenografts (PDX) of GC. After successful establishing the PDX model, NSG mice received intratumoral injections of AAV9‐sgHCP5‐132aa virus prepared with the CRISPR/Cas9 technology, or AAV9‐Ctrl‐gRNA as a control. Due to vector capacity limitations, spCas9 and sgRNA were delivered using separate vectors, requiring the co‐administration of AAV viruses expressing sgRNA and AAV9‐mini‐CMV‐Cas9 (Figure 7J). Simultaneously, to further analyze the sensitivity of GC patients with different expression levels of HCP5‐132aa to ferroptosis inducers, we established PDX models using GC tissues from two patient groups with varying HCP5‐132aa expression level. Patient 1 exhibited high expression, whereas Patient 2 showed relatively low expression of HCP5‐132aa (Figure 7J; Figure S8B Supporting Information). After six weeks of observation, subcutaneous tumors in mice injected with AAV‐sgHCP5‐132aa showed significantly reduced size, volume and weight compared to controls, while erastin‐induced ferroptosis also significantly decreased tumor size compared to controls (Figure 7K–M). Furthermore, GC‐PDX with low HCP5‐132aa expression required less time to reduce the tumor volume by 50% after erastin treatment than the high HCP5‐132aa group, suggesting that GC tumors with low HCP5‐132aa expression levels are more sensitive to erastin therapy than those with high levels (Figure 7N). Concurrently, successful establishment of the PDX model was confirmed through H&E staining and immunohistochemistry, showing significantly reduced expression of Ki‐67 and HCP5‐132aa in the HCP5‐132aa knockout group, with induction of ferroptosis upon HCP5‐132aa knockout (Figure S8C Supporting Information). Additionally, western blotting analysis also confirmed that the knockout of HCP5‐132aa in GC‐PDX models or treatment with erastin in groups with relatively low HCP5‐132aa expression in GC‐PDX models facilitated enhanced ferroptosis, further validating the aforementioned observations (Figure S8D,E Supporting Information). In summary, our findings affirm that the reduction of HCP5‐132aa levels in vivo can induce ferroptosis and thereby inhibit tumor growth in GC, suggesting that HCP5‐132aa may be a promising therapeutic target for GC (**Figure** **8**).

## 3 Discussion

Over the past few decades, the Human Genome Project, the ENCODE Project, and the rapid development of multi‐omics technologies have allowed us to gain a deeper understanding of translation at the whole transcriptome level. However, the predominant focus of research has historically centered on protein‐coding genes. In recent years, the discovery of the translational potential of lncRNAs and their encoded new proteins or peptides has provided new perspectives and insights for exploring the underlying mechanisms responsible for the occurrence and progression of cancers and other diseases. For instance, the polypeptide ATMLP produced by lncRNA AFAP1‐AS1 triggers malignancy in non‐small cell lung cancer by binding to NIPSNAP1 and inhibiting its translocation from the inner to the outer mitochondrial membrane, thereby antagonizing NIPSNAP1‐mediated regulation of cellular autolysosome formation.^[^ ^52^ ^]^ Besides, Zhang et al. reported that LINC00673 encoded novel protein RASON is a key regulator of oncogenic KRAS signaling in pancreatic ductal adenocarcinoma, and could bind directly to KRASG12D/V and inhibit intrinsic and GAP‐mediated GTP hydrolysis, thereby maintaining KRASG12D/V in a highly active state of GTP binding.^[^ ^53^ ^]^ In this study, we performed an integrative analysis combining transcriptomic and translatomic data, identifying HCP5 as an lncRNA associated with GC that possesses coding potential. Further MS, point mutation and immunoblotting assays determined that HCP5 could translate a novel protein that we named HCP5‐132aa in GC cells and tissues.

HCP5 has been found to be over‐expressed in a variety of tumors, including GC and esophageal squamous cell carcinomas, and to possess cancer‐promoting functions by exerting competing endogenous RNA (ceRNA) activity or interacting with proteins such as UTP3.^[^ ^20^, ^54^, ^55^ ^]^ Here, we have demonstrated that the new protein HCP5‐132aa produced by HCP5 is upregulated in GC tissues and cells, and that high expression of HCP5‐132aa is associated with poor prognosis in patients with GC. As HCP5‐132aa is a new and uncharacterized protein in GC, we explored its biological roles and underlying molecular mechanisms in‐depth. Our findings from loss‐and gain‐of function and rescue assays revealed that HCP5‐132aa exerts tumor‐promotion functions through facilitating cell proliferation and inhibiting apoptosis both in vitro and in vivo. Further immunoprecipitation combined with MS analysis indicated that HCP5‐132aa could interact with YBX1 in GC cells. Mechanistically, the interaction of HCP5‐132aa with YBX1 does not affect its protein expression level, but it does enhance the interaction of YBX1 with its counterpart protein ELAVL1. YBX1, a multifunctional RNA binding protein, is implicated in various cellular processes under physiological conditions and tumorigenesis through regulating gene transcription in the nucleus and RNA translation and stability in the cytoplasm.^[^ ^56^, ^57^ ^]^ Recently, YBX1 has been identified as a novel RNA m^5^C modification reader that binds directly to m^5^C methylated RNAs and maintains their stability.^[^ ^58^ ^]^ m^5^C is a critical post‐transcriptional modification on mammalian RNA, which can be catalyzed by RNA methyltransferases such as NSUN2 and DNMT2. Aberrant modifications of mRNA m^5^C have been reported to be involved in the development and drug resistance of esophageal squamous cell carcinoma, GC, and non‐small cell lung cancer.^[^ ^41^, ^59^, ^60^, ^61^ ^]^

To further explore whether the HCP5‐132aa/YBX1/ELAVL1 complex contributes to GC by regulating mRNA stability via recognizing the m^5^C site, we analyzed RNAseq data combined with YBX1 iCLIP data and found that most of HCP5‐132aa and YBX1 co‐regulated genes expression levels were decreased under the knockdown of YBX1 and knockout of HCP5‐132aa. Subsequent validation of the top target genes drew our attention to SLC7A11 and G6PD, which were most significantly downregulated, and their mRNA stability was also affected following deletion of HCP5‐132aa and YBX1 expression. Interestingly, bioinformatic analysis and experimental validation confirmed that YBX1 could directly bind to the m^5^C site in the 3′UTR of G6PD and SLC7A11 mRNA and stabilize their stability by recruiting ELAVL1, and this regulation process is influenced by HCP5‐132aa. SLC7A11 and G6PD are important regulators involved in ferroptosis, and the results of our GO analysis show that HCP5‐regulated genes are closely associated with ferroptosis. Ferroptosis is a form of regulated cell death triggered by iron‐dependent phospholipid peroxidation, which has been shown to be involved in the development of various diseases, such as cancers, and targeting ferroptosis promises to be an effective strategy for the treatment of cancers.^[^ ^62^ ^]^ Our subsequent results indicated that HCP5‐132aa intervention also affects the ferroptosis of GC cells as well as depletion of SLC7A11 and G6PD. Consistent with our findings, a recent study by Wang et al. also demonstrated that HCP5‐132aa could regulate ferroptosis in triple‐negative breast cancer, but did not clarify the underlying mechanism.^[^ ^63^ ^]^ Our findings prove that HCP5‐132aa enhances the interaction of YBX1 with ELAVL1 and thus maintains the stability of SLC7A11 and G6PD MRNAs by recognizing the m^5^C sites in the 3′UTR of their mRNAs, ultimately inhibiting ferroptosis in GC cells.

In addition to their functional roles in tumorigenesis, there is evidence showed that lncRNA encoded novel proteins or peptides can serve as intervention targets for cancer therapy and be deployed as new neoantigens for cancer immunotherapy.^[^ ^9^ ^]^ For instance, Thangue and colleagues found that a number of MHC class I protein‐associated peptides were derived from small ORFs in lncRNAs, and these peptides can drive a robust antigen‐specific CD8 T lymphocyte response, which translates into a marked delay in tumor growth.^[^ ^64^ ^]^ What's more interesting is that targeting knock out of ASAP encoded by LINC00467 with CRISPR/Cas9‐based strategy can efficiently suppresses the growth of colon cancer PDX.^[^ ^65^ ^]^ Given the function of HCP5‐132aa in promoting the proliferation of GC cells, we designed a targeting elimination strategy based on CRISPR/Cas9 system toward HCP5‐132aa. In fact, we gained evidence that silencing of HCP5‐132aa repressed the growth of GC PDXs in vivo, which suggest HCP5‐132aa as a potential therapeutic target for GC.

In summary, our findings highlight the oncogenic function of HCP5 derived protein HCP5‐132aa in GC. HCP5‐132aa is characterized as a novel regulator of ferroptosis to promote cell proliferation of GC by ensuring the stability of downstream genes associated with YBX1. These evidences provide new insights for the investigation of lncRNA encoded proteins in GC, and indicate a powerful therapeutic approach by pharmacological manipulation of HCP5‐132aa for GC.

## 4 Experimental Section

### Cell Culture

The human gastric mucosal cell line (GES‐1), GC cell lines (NCI‐N87, AGS, HGC‐27) and human embryonic kidney 293T cells (HEK‐293T) were purchased from National Collection of Authenticated Cell Cultures (Shanghai, China). GC cell line MKN‐45 were obtained from China Infrastructure of Cell Line Resources. All GC cell lines were cultured in RPMI 1640 medium (Gibco, USA), while GES‐1 and HEK‐293T cells were cultured in DMEM medium (Gibco, USA). 10% fetal bovine serum and 1x penicillin‐streptomycin (Invitrogen, USA) were added to cell culture medium and the cells were maintained in a humidified incubator at 37 °C with 5% CO_2_. All cells used in this study were monitored for mycoplasma contamination and were authenticated by short tandem repeats (STR) sequencing.

### Tissue Samples

Fresh frozen primary GC tissues and their matched adjacent nontumoral stomach tissues, which had been pathologically verified, were collected from 70 GC patients with at the Yantai Yuhuangding Hospital. All patients received no other treatment prior to tumor resection. After surgical resection, the specimens were immediately frozen and stored at −80 °C. Informed consent was obtained from each patient, and the collection of these tissue samples was approved by the Ethics Committee of Nanjing Medical University. Second, the tissue microarray chips containing GC tissues from 80 GC patients were purchased from AiFang biological (AF‐StcSur2201), and the related clinicopathological and survival information was also provided.

### Total RNA Extraction and Quantitative Real‐Time PCR (RT‐qPCR)

TRIzol reagent (Invitrogen, USA) was using for extracting total RNA from all cell lines and tissues used in this study. The concentration and purity of all samples were subsequently measured via NanoDrop2000 Spectrophotometer. Complementary DNA was synthesized using the Reverse Transcription Kit (TransGen, China), and the cDNA was used for quantitative real‐time PCR using Hieff qPCR SYBR Green Master Mix (YEASEN, China) according to the manufacturer's instructions. GAPDH was used as an internal control. The relative expression was calculated by the 2^−ΔΔCT^ method. The primers used are listed in Table S2 (Supporting Information).

### Purification of Ribosome‐Bound RNA

The ribosome‐bound RNA was purified as previously described.^[^ ^66^ ^]^ Briefly, cells were treated with 100 µg mL^−1^ cycloheximide for 15 min at 37 °C and then washed twice with ice‐cold PBS buffer. These cells were lysed with ice‐cold MCB polysome lysis buffer and the cell lysates were centrifuged to remove cell debris at 12 000 g for 30 min at 4 °C. The supernatant was loaded onto top of 15% to 50% sucrose gradients and separated by ultracentrifugation with a SW41 rotor (Beckman) at 180 000 g for 4 h at 4 °C. The ribosome‐bound RNAs were harvested using Trizol reagent and subsequently used for by RT‐qPCR.

### Western Blotting Analysis

Tissue or cell proteins were extracted with RIPA buffer (Beyotime, China) containing protease inhibitor. The samples were separated by sodium dodecyl sulfate polyacrylamide gel electrophoresis (SDS‐PAGE) using an equal amount per sample, followed by transfer onto a PVDF membrane (Bio‐Rad, USA). The membrane was blocked using 5% non‐fat milk. After incubation with primary antibodies at 4 °C overnight, the PVDF membranes with secondary antibodies at room temperature were incubated for 1 h. The immunoreactive signals detection was performed by Immobilon ECL substrate (Millipore, Germany). Image Lab software (Bio‐Rad, USA) was used to analyze the imprinted images, and then ImageJ was used for gray value density quantification. Information on the antibodies is listed in Table S4 (Supporting Information).

### In Vitro Transcription/Translation

Using genomic DNA as a template, the full‐length sequence of lncRNA HCP5 was amplified with primers containing the T7 promoter sequence and cloned into the pcDNA‐3.1 vector. Subsequently, linearized DNA containing the T7 promoter was utilized as a template for in vitro transcription using the RiboTM RNAmax‐T7 Transcription Kit (ribobio) following the manufacturer's instructions, and the resulting product was purified using Purification Assistant A/B. Following this, in vitro translation experiments were conducted using the Flexi Rabbit Reticulocyte Lysate System (Promega) by preparing the transcription products with translation reaction components according to the instructions. The reaction mixture was then incubated at 30 °C for 90 min in the presence of 35S‐methionine. After incubation, the translation products were mixed with an appropriate volume of 5× loading buffer, denatured at 95 °C for 10 min, separated by SDS‐polyacrylamide gel electrophoresis (PAGE), and detected by autoradiography.

### Immunofluorescence Staining

GC cells were seeded into a 24‐well plate containing cell slides. After stable adherence and growth of the cells, they were fixed with 4% paraformaldehyde solution, permeabilized with 0.5% Triton X‐100 for 10 min and blocked with 5% BSA for 1 h. In order to assess the initiation codon activity of HCP5‐132aa ORF15, GC cells were transfected with the respective plasmids (vector, HCP5‐132aa‐Flag and HCP5‐132aa mut‐Flag), and immunostaining was performed with Flag (1:100) primary antibodies followed by suitable Goat Anti‐Rabbit IgG(H+L) Cy3 secondary antibodies. For protein localization analysis, immunostaining was conducted using primary antibodies against HCP5‐132aa (1:200), YBX1 (1:200), and ELAVL1 (1:200) followed by appropriate secondary antibodies. The nuclei were stained with 4′,6‐diamidino‐2‐phenylindole (DAPI) (Thermo Fisher Scientific). The acquisition of images was carried out using the confocal microscope (OLYMPUS, FV1200).

### Oligonucleotide Transfection

The siRNA interference sequence used in this study were synthesized by GenePharma (GenePharma Corporation, Shanghai, China). A nontargeting scrambled siRNA was used as a control. In brief, different GC cells were inoculated in 6‐well plates and cultured to ≈80% before transfection. Then siRNAs were transfected into cells according to the manufacturer's protocol using the Lipofectamine 2000 transfection kit (Invitrogen, USA). The knockdown efficiency and specificity of all siRNAs were validated by RT‐qPCR. The siRNAs used in this study are listed in Table S3 (Supporting Information).

### Plasmid Constructs

The lncRNA HCP5 ORFs‐Flag, HCP5‐132aa‐EGFP, YBX1‐HA, ELAVL1‐His, SLC7A11, and G6PD overexpressed plasmids were generated as previously described.^[^ ^67^ ^]^ The mutated plasmids (HCP5‐13aa mut‐Flag, GFPmut, HCP5‐132aa mut‐GFP, HCP5‐132aa mut‐GFPmut) were generated using a Mut Express II Fast Mutagenesis Kit V2 (Vazyme, China), in which the start codons of the HCP5‐13aa and GFP were mutated to ATT. To generate truncated plasmids, the truncated forms of HCP5‐132aa, YBX1, and ELAVL1 were obtained by PCR amplification and subcloned into pcDNA3.1. The sgRNAs sequence targeting HCP5‐132aa were designed and cloned into lentiCRISPA V2 (lentiCRISPR‐sgRNA‐HCP5). All constructs were confirmed by DNA sequencing at Sequencing.

### Construction of Cell Lines with Stable Expression

For lentivirus infection to generate stable cell lines, HEK‐293T cells were transfected with HCP5‐132aa‐Flag and lentiCRISPR‐sgRNA‐HCP5, along with the packaging and envelope plasmids psPAX2 and PCMV‐VSV‐G at a 4:3:2 ratio respectively using Lipofectamine 2000 (Life Technologies, USA) according to the manufacturer's instructions. Virus particles were harvested 48 h after transfection, and concentrated by 0.45 µm filter membrane. MKN‐45, NCI‐N87, and HGC‐27 were infected with lentivirus plus 6 µg mL^−1^ polybrene (Sigma‐Aldrich, USA). After 48 h, the lentivirus‐infected cells were selected with 4 µg ml^−1^ puromycin (Sangon Biotech, China). The knockout efficiency of HCP5‐132aa was tested by sang sequencing, and the expression of HCP5‐132aa protein was verified by western blotting.

### Cell Viability Assay

GC cells were inoculated in 96 well plates (3000 cells per well) and allowed to adhere for 12 h at 37 °C in medium. After treatment, cells were further incubated with the CCK‐8 reagent (Vazyme, Nanjing, China) at 10% concentration in complete medium for 2–4 h. Then the absorbance at 450 nm was measured. The survival of genotoxin‐exposed cells was determined by relating the absorbance to that of an untreated control.

### Colony Formation Assay

Indicated GC cells were seeded in a 6‐well plate at a density of 1000 cells per well. The cells were cultured for 10–14 days. Cells were fixed using 4% paraformaldehyde for 20 min and stained with 0.5% crystal violet for 10 min. The colony numbers were counted under a microscope.

### 5‐ethynyl‐2′‐ eoxyuridine (EdU) Assay

The cell proliferation was analyzed by using the 5‐ethynyl‐2′‐ deoxyuridine (EdU) Cell Proliferation Kit (Beyotime, China, C0078S). Briefly, the transfected cells were incubated with EdU solution (50 µm) for 2 h in 24‐well plates (1 × 10^5^ well^−1^), and then fixed with 4% paraformaldehyde for 30 min at room temperature. Subsequently, the permeable solution was incubated at room temperature for 15 min, and the configured Click reaction solution was added for 30 min and incubated away from light. Finally, Hoechst 33 342 was added to stain the nucleus for 10 min in a dark room at room temperature, and EdU‐positive cells were observed and counted with a fluorescence microscope.

### Co‐Immunoprecipitation (Co‐IP) and Mass Spectrometry (MS)

Co‐immunoprecipitation assay using the Pierce Co‐Immunoprecipitation Kit (Thermo Fisher Scientific). In short, following the manufacturer's protocol, GC cells were treated with IP lysis buffer for a duration of 4 h. After the treatment, the cells were centrifuged at a speed of 12000 g for 10 min, and the resulting supernatant was collected. Afterward, primary antibody was added to the supernatant and incubated overnight at a temperature of 4 °C. Subsequently, Protein A/G beads were introduced and incubated for 2 h at room temperature. The beads were isolated utilizing a magnetic rack, followed by washing and a subsequent round of separation. The samples were then subjected to SDS‐PAGE for the purpose of separation. Following the separation, the distinct bands were visualized using both the silver staining method and western blotting. Furthermore, for protein identification, LC‐MS/MS analysis was conducted in accordance with the previously described.^[^ ^68^ ^]^ Subsequent to the immunoprecipitation assay, the proteins underwent SDS‐PAGE gel electrophoresis, and the corresponding protein bands were excised to enable LC‐MS/MS analysis. This analytical approach facilitated the identification of peptide sequence mixtures derived from the excised gel.

### Proximity Ligation Assay (PLA)

PLA can detect physical contacts between two proximate protein molecules, revealing the location and extent of protein interactions within cells. For this experiment, GC cells stably expressing HCP5‐132aa‐Flag OE were seeded in a 12‐well plate with 12 mm diameter coverslips and allowed to adhere. After stabilization, cells were washed twice with PBS and fixed with 4% paraformaldehyde for 10 min, followed by permeabilization with 0.5% Triton X‐100 for 10 min. The cells were then incubated in Duolink blocking solution at 37 °C for 60 min; primary antibodies (anti‐Flag mouse monoclonal and anti‐YBX1/ELAVL1 rabbit monoclonal) were diluted in Duolink antibody diluent and incubated overnight at 4 °C. PLUS and MINUS PLA probes were diluted 1:5 in Duolink antibody diluent and applied to the coverslips, followed by a 60 min incubation at 37 °C. Ligation buffer was then added, and the samples were incubated for another 30 min at 37 °C. Amplification buffer was prepared and added to the samples, which were then incubated at 37 °C for 100 min. The samples were coverslipped using a mounting medium containing DAPI for 15 min. The localization of HCP5‐132aa and YBX1/ELAVL1 in GC cells was observed using a confocal laser scanning microscope.

### GST Pull Down

Plasmids expressing the recombinant proteins (pGEX‐YBX1‐GST, pET‐ELAVL1‐His, and pET‐HCP5‐132aa‐His) were constructed and transformed into BL21 cellsc for cultivation until reaching the logarithmic growth phase, followed by induction of protein expression with IPTG. The cell pellets were then subjected to sonication in NETN buffer (20 mm Tris‐HCl pH 8.0, 200 mm NaCl, 0.5mm EDTA, 0.5% NP‐40) containing 1 mm PMSF and subsequently centrifuged at 13000 g for 30 min at 4 °C. The recombinant proteins tagged with GST or His were purified using BeyoGold GST‐tag Purification Resin (Beyotime, China, P2262) or BeyoGold His‐tag Purification Resin (Beyotime, China, P2229S), respectively. The purified GST‐tagged protein was incubated with the His‐tagged protein in 500 µL binding buffer supplemented with the corresponding GST resin at 4 °C for 3 h with gentle rotation. Subsequently, the resin was washed three times with NETN buffer. The resin was then boiled with 2× SDS loading buffer, and the protein enriched in the resin was detected via western blotting analysis.

### RNA‐Binding Protein Immunoprecipitation (RIP)

The GC cells for testing were harvested and suspended in RIP lysis buffer (Millipore, MA, USA) supplemented with DNase I and RNAase inhibitors. Subsequently, incubate the cell suspension on ice for 15 min at 4 °C, followed by centrifugation at 12000 rpm for 10 min to obtain the supernatant. Simultaneously, the protein A/G magnetic beads (Life Technologies) were then incubated with the anti‐YBX1 or anti‐ELAVL1 antibody for the RIP group or mouse IgG isotype control for the IgG group at room temperature for 2 h. Following this, the antibody‐bead complexes were added to the cell lysate and incubated overnight at 4 °C. The magnetic rack was utilized for the collection of magnetic beads, subsequent removal of supernatant, and cleaning of the beads with RIP buffer. Finally, the RNA was extracted by TRIzol (Invitrogen) and determined by RT‐qPCR. The recovery rate of specific transcripts was calculated by comparing the relative mRNA abundance of target genes in RIP group and input group, and the recovery rate of specific transcripts was calculated by abundance, and the data of each group was normalized by GAPDH level.

### RNA Stability Analysis

GC cells at 80% confluence were exposed to a concentration of 5 µg ml^−1^ actinomycin D and harvested at specified time intervals. Total RNA was extracted using the TRIzol reagent (Invitrogen) and the mRNA levels of SLC7A11 and G6PD were assessed via RT‐qPCR. A reference gene GAPDH was used to normalize the differences between samples. The decay rate and half‐life of the mRNA were calculated using established methodologies as described in previous studies.^[^ ^69^ ^]^

### Methylated RNA Immunoprecipitation (MeRIP)

The MeRIP assay was conducted to evaluate the m^5^C modification of SLC7A11 and G6PD using the Magna MeRIP Kit (Millipore) in accordance with the manufacturer's instructions. In brief, total RNA was fragmented using an ultrasonicator. Afterward, protein A/G agarose beads were incubated with either an anti‐m^5^C antibody or a control IgG antibody at 4 °C for a duration of 4 h. The sheared RNAs were then incubated with the antibody‐bound beads in IP buffer (150 mm NaCl, 0.1% NP‐40, 10 mm Tris‐HCl, pH 7.4, 0.4 U µl^−1^ RNase inhibitor) for 6 h at 4 °C. The m^5^C‐methylated RNAs that were immunoprecipitated were isolated using a magnetic stand and subsequently recovered through proteinase K digestion. The RNA was further extracted using TRIzol (Invitrogen) and subjected to RT‐qPCR analysis to assess its level. The enrichment of m^5^C in each sample was calculated by normalizing to the input.

### RNA Methylation Assay

The RNA methylation assay was conducted using the EZ RNA Methylation Kit (ZYMO, Irvine, USA) following the manufacturer's instructions. To carry out the RNA conversion, 130 µl of RNA conversion reagent was added to the PCR tube containing a 20 µl RNA sample. The PCR tube was then placed in a thermal circulator and incubated at 70 °C for 5 min, followed by 54 °C for 45 min, and finally at 4 °C for 20 h. Subsequently, 250 µl of RNA binding buffer and 400 µl of ethanol were sequentially added to the mixture, which was then centrifuged at maximum speed for 1 min. The supernatant was discarded. Next, 200 µl of RNA desulphurization buffer was added to the column and left at room temperature for 30 min, followed by centrifugation at maximum speed for 1 min, and the filtrate was discarded. The column was washed twice with 400 µl of RNA Wash Buffer. Finally, the RNAs were eluted in 10 µl of RNase‐free water. To confirm the methylation site, the sulfite‐treated mRNA was subjected to amplification using a specific primer, and the resulting PCR products were subsequently sequenced using the Sanger sequencing method.

### Measurements of GSH/GSSG Ratio

GSH and GSSG assay kits (Solarbio, BC1175 and BC1180) were used to measure the GSH/GSSG ratio. The cells were washed with ice‐cold PBS and then lysed in GSH assay buffer. GSH levels were determined following the manufacturer's instructions. The measurement of GSH involved a kinetic assay, in which catalytic amounts (nmoles) of GSH caused a continuous reduction of 5,5′‐dithiobis (2‐nitrobenzoic acid) to 5‐thio‐2‐nitrobenzoic acid. The rate of the reaction was directly proportional to the concentration of glutathione. The spectrophotometric measurement of the yellow product (5‐thio‐2‐nitrobenzoic acid) was performed at 412 nm.

### Measurements of NADP^+^/NADPH Ratio

NADP+/NADPH ratio levels were analyzed using an NADP/NADPH Assay Kit (Solarbio, BC1100). GC cells were lysed using an NADP/NADPH extraction buffer, and the sample was subjected to extraction using both acidic and alkaline extraction solutions to extract NADP^+^ and NADPH. NADP^+^ and NADPH levels were determined following the manufacturer's instructions. NADPH reduces the oxidized form of thiazolyl blue to formazan through the diaphorase activity of PMS. The absorbance at 570 nm was measured to determine the NADPH content. NADP^+^ content was detected by reducing NADP^+^ to NADPH using glucose 6‐phosphate dehydrogenase, and then colorimetric readings were measured at 570 nm by spectrophotometry.

### Lipid ROS Measurement

Lipid ROS levels were measured using the C11‐BODIPY fluorescent probe (HY‐D1301, MCE). The C11‐BODIPY probe is known for its excellent photostability, low fluorescence artifacts, and efficient cell membrane permeability. The GC cells to be analyzed were cultured in six‐well culture plates at a density of 2.5 × 10^5^ cells per well and incubated overnight. Subsequently, the cells were stained with 2 mL of medium containing 5 µm BODIPY‐C11 and incubated at 37 °C in a dark environment for 20 min. To remove any excess staining solution, the cells were washed twice with PBS and then re‐suspended in 500 µL of fresh PBS. Following the manufacturer's instructions, the appropriate detection working solution was added, and the levels of oxidative stress in the supernatant were determined using a fluorescent microplate reader.

### Malondialdehyde (MDA) Assay

The relative Malondialdehyde (MDA) concentration in cells was measured using the MDA assay kit (Nanjing Jiancheng Bioengineering Institute, A003‐1‐2). MDA can react with thiobarbituric acid (TBA) to form a red product, with a maximum absorption peak at 532 nm. In summary, the cells under investigation were treated with MDA lysis buffer and lysed on ice. After collecting the supernatant, TBA reagent was added and incubated at 95 °C for 60 min. The reaction mixture was then cooled to room temperature in an ice bath. Finally, 200 µL of the MDA‐TBA reaction compound was transferred to a 96‐well plate, and the absorbance was measured at 593 nm to calculate the MDA content in the sample.

### Iron Assay

Intracellular iron levels (Fe^2+^) were determined with a standard iron assay kit (Abcam, ab83366) according to the manufacturer's protocol. Briefly, the cells were harvested and washed with cold PBS, followed by homogenization in five times the volume of ice‐cold iron assay buffer. The homogenate was then centrifuged at 13000 × g for 10 min at 4 °C to remove insoluble substances. The supernatant was collected, and iron reducer was added to each sample before mixing. The samples were then incubated at room temperature for 30 min. Subsequently, 100 µL of iron probe was added to each sample and mixed thoroughly, followed by an additional incubation in the dark at room temperature for 1 h. Finally, the absorbance at 593 nm was promptly determined with the microplate reader.

### Transmission Electron Microscopy

The GC cells designated for testing were harvested and combined with 8% paraformaldehyde in a 1:1 ratio for a minimum of 1 h. Following that, 20 µL of the fixed sample was applied to a Formvar‐stabilized 230‐mesh carbon support film and allowed to incubate for 5 min at room temperature. The copper grid was soaked in 20 µL of 3% phosphotungstic acid and subsequently air‐dried for 5 min at room temperature. Afterward, the copper mesh was removed, and any excess liquid droplets on the edge were absorbed using filter paper. Finally, the mitochondria within the GC cells were visualized utilizing the Olympus EM208S transmission electron microscopy.

### RNA Pull‐Down Assay

Biotin‐labeled RNA oligonucleotides containing C or m^5^C were synthesized by GeneScript (China). RNA affinity purification was performed using a PierceTM Magnetic RNA‐Protein Pull‐Down kit (Thermo Fisher Scientific) according to the manufacturer's instructions. In brief, each 1 nmol of RNA oligonucleotide was added to a binding buffer containing 50 µL of streptavidin magnetic beads and incubated at 4 °C for 4 h. Subsequently, the RNA bait‐conjugated streptavidin magnetic beads were incubated with nuclear extracts prepared from stable knockout or overexpressing GC cells with HCP5‐132aa in the binding buffer at 4 °C overnight. The elution of these beads was performed by adding 30 µL of protein loading buffer followed by boiling for 5 min. The eluted mixtures were then subjected to western blotting analysis. Biotin‐labeled RNA oligonucleotides sequences were provided in Table S3 (Supporting Information).

### Chromatin Immunoprecipitation (ChIP) Assay

The ChIP assay was conducted by using the EZ ChIP Kit (Merck Millipore, Darmstadt, Germany) in accordance with the manufacturer's guidelines. In brief, cells were treated with 1% formaldehyde for 10 min to enable cross‐linking. After quenching the reaction with glycine, cell pellets were lysed using a lysis buffer and subjected to 5 min of sonication. The resulting lysates underwent centrifugation, followed by an overnight incubation at 4 °C with anti‐TFAP2A. Immunocomplexes were then captured by employing 30 µl of protein A/G sepharose and rotating the samples at 4 °C for 2 h. Following a series of washes, the complexes were eluted using an elution buffer. The obtained supernatant was subjected to overnight reverse‐cross‐linking at 65 °C and subsequent proteinase K treatment for 4 h at 55 °C. The ChIP and input DNA samples were consequently purified and subjected to RT‐qPCR analysis. The calculated enrichment value was normalized against the input and presented as a ratio to IgG. Primer sequences were as follows: promoter region of HCP5, forward 5′‐ CTGTAAGGCAGACACCCTTG ‐3′, and reverse 5′‐CTGCTGGAGTGTCCCATGA ‐3′.

### Dual‐Luciferase Reporter Assay

The construction of chimeric firefly luciferase reporter plasmids was performed following the previously described protocol.^[^ ^70^ ^]^ Briefly, the 3′UTR regions of SLC7A11 or G6PD mRNA containing m^5^C sites were cloned into the pmir‐GLO plasmid. For the mutant reporter constructs, the cytosine (C) at the m^5^C site was replaced with adenine (A). Stable HCP5‐132aa knockout GC cells or HEK‐293T cells were seeded in 24‐well plates in three rounds until reaching 80% confluency. Subsequently, the reporter plasmids pMIR‐SLC7A11‐WT, pMIR‐SLC7A11‐mut, pMIR‐G6PD‐WT, and pMIR‐G6PD‐mut were individually co‐transfected with YBX1‐WT or YBX1‐Mut expression vectors using Lipofectamine 2000 (Invitrogen). Alternatively, the promoter sequences of HCP5 were amplified from GC cell genomic DNA using PCR and inserted into the pGL3‐basic expression vector. To create HCP5‐Mut plasmids, the binding site (GCCTGGGGC) was substituted with (GGATTGACC) using a Mut Express II Fast Mutagenesis Kit V2 (Vazyme, China). Subsequently, GC cells were seeded in individual wells of a 24‐well plate and co‐transfected with or without the TFAP2A expression vector using Lipofectamine 2000. After 48 h, the luciferase reporter gene assay kit (YEASEN, Shanghai, China, 11402ES60) was employed to measure the relative luciferase activity in accordance with the manufacturer's instructions.

### Immunohistochemistry Staining

IHC was employed to evaluate the expression of the HCP5‐132aa and Ki‐67 protein in the GC tissues. The tissue sections underwent dehydration and rehydration processes, followed by antigen retrieval using sodium citrate buffers. Next, 5% goat serum was applied and incubated at room temperature for 30 min. Subsequently, rabbit anti‐HCP5‐132aa and anti‐Ki‐67 antibodies were added and incubated overnight at 4 °C, followed by incubation with a goat anti‐rabbit secondary antibody at room temperature for 30 min. Afterward, DAB was utilized for color development, which lasted ≈1 min. The reaction was terminated by rinsing with running water, and the tissue sections were dehydrated using a gradient of alcohol, cleared with xylene, and sealed with neutral glue. Finally, the expression levels of HCP5‐132aa and Ki‐67 were observed under an optical microscope. HCP5‐132aa protein level scores were evaluated using the previously described semi‐quantitative method. Scores ≤4 indicated low HCP5‐132aa levels (low) in GC tissues, while scores >4 indicated high HCP5‐132aa levels (high) in GC tissues.

### AAV9 Virus Production and Purification

Insert the HCP5‐132aa gRNA sequence (5′‐ GAGACCAGCGGGTGAGAAGtgg‐3′) into the AAV vector (AAV9‐sgHCP5‐132aa) and use this construct along with a vector expressing Cas9 under the control of a CMV promoter for AAV9 virus packaging. Send the AAV9‐sgHCP5‐132aa and AAV9‐Ctrl‐gRNA donor vectors to PackGene Biotech (Guangzhou, China) for virus packaging and production. The control gRNA does not target any specific gene. Package all viral vectors into AAV9. Purified aliquots of AAV9 were stored at −80 °C. As per the company's report, the titers of AAV9‐sgHCP5‐132aa, AAV9‐mini‐cmv‐spCas9, and AAV9‐Ctrl‐gRNA were 1E+13GC/mL respectively.

### Tumor Xenograft In Vivo

The 4‐6‐week‐old BALB/C male nude mice were purchased from the Animal Center of Nanjing Medical University, and the animal experiments were conducted in strict accordance with the management and use rules formulated by the Experimental Animal Committee of Nanjing Medical University. In subcutaneous tumor transplantation model of nude mice, 1.5 × 10^6^ GC cells from each group (Ctrl, HCP5‐132aa KO, KO+HCP5‐132aa, and KO+HCP5‐132aa mut) were resuspended in 0.1 ml PBS and subsequently injected into the subcutaneous back of nude mice. The tumor volume was measured and observed regularly. After 5–8 weeks, the nude mice were sacrificed and the tumors were fixed with 4% paraformaldehyde. Then H&E staining was performed to evaluate the degree of tumor necrosis, and Ki‐67 and HCP5‐132aa antibodies were used to detect its expression by immunohistochemistry.

### Patient‐Derived Xenograft Model

Four‐week‐old NSG mice were purchased from GemPharmatech (Nanjing, China) and housed under the same conditions with nude mice (BALB/c). Ethical approval was approved by the Ethics Committee of Nanjing Medical University (Nanjing, China). Fresh tumor samples from two patients with GC were randomly selected, the necrotic tissue and adipose tissue were removed, and the samples were divided into ≈2 mm^3^ sections. Mice were anesthetized, and small tissue pieces were then inoculated beneath the skin, with subsequent suturing of the wounds. Tumor volume was monitored at 3‐day intervals, and when it reached a diameter of 1 cm, tumors of approximately equal volume (≈2mm^3^) were subcutaneously implanted into 4–5 weeks old NSG mice. Once the tumor size reached ≈200 mm^3^, all mice were randomly assigned into either the Ctrl or HCP5‐132aa KO group and treated with intratumor injection of the corresponding lentivirus. Mix the viruses expressing gRNA and Cas9 in a 1:2 ratio, then inject 30 µl of the mixed virus (totaling 1E+11GC) via in situ injection into the GC‐PDX model. Tumor volume was measured once a week. Additionally, two groups would be established to receive injections of DMSO and erastin, respectively. After 8 weeks, all mice were sacrificed, and subcutaneous tumors were subjected to H&E and IHC analysis.

### Statistical Analysis

Experimental data were analyzed using SPSS 20.0, GraphPad, Prism 7.0 and ImageJ software. Data were presented as mean ± SD unless otherwise indicated from 3 independent experiments. Differences between the two groups were assessed using two‐tailed unpaired Student's t‐tests or Mann‐Whitney U‐tests. Two‐way ANOVA was employed to analyze the significance of the growth curves. Kaplan‐Meier method with the log‐rank test was utilized to determine the survival curves. Sample sizes and significance were shown in corresponding figure legends. *p < 0.05, **p < 0.01 and *** *p* < 0.001 were considered statistically significant.

## Conflict of Interest

The authors declare no conflict of interest.

## Author Contributions

Q.H.L., Y.L.C., M.S., and X.H.L. performed conceptualization; Q.H.L. and G.Q.G. performed methodology; Y.L.C., L.L., and M.S. performed investigation; Q.H.L. and G.Q.G. performed visualization; M.S. and X.H.L. performed funding acquisition; Z.H.Z, J.H.G., X.K.G., Y.M.H., and Q.N.L. performed project administration; L.L., H.Y.L., J.H.G., and Y.M.H. performed supervision; Q.H.L., G.Q.G., Y.L.C., and M.S. wrote the original draft; Q.H.L., X.H.L., and M.S. wrote, reviewed and edited.

## Supporting information



Supporting Information

Supporting Information

## Data Availability

Research data are not shared.
