# Supplementary material for: HCP5 Derived Novel Microprotein Triggers Progression of Gastric Cancer through Regulating Ferroptosis
Source: Adv Sci (Weinh). 2024 Oct 24;11(46):2407012. doi: 10.1002/advs.202407012 (PMC11633528; doi:10.1002/advs.202407012)

ADVANCED SCIENCE Open Access

Supporting Information

for *Adv. Sci.*, DOI 10.1002/advs.202407012

HCP5 Derived Novel Microprotein Triggers Progression of Gastric Cancer through Regulating Ferroptosis

Qiuhui Li, Guoqing Guo, Yuli Chen, Lu Lu, Hanyang Li, Zihan Zhou, Jiahao Guo, Xiongkang Gan, Yanming Hu, Qiunuo Li, Ming Sun and Xianghua Liu**

Supplementary information

HCP5 derived novel microprotein triggers progression of gastric cancer through regulating ferroptosis

Authors: *Qiuhui Li#, Guoqing Guo#, Yuli Chen#, Lu Lu, Hanyang Li, Zihan Zhou, Jiahao Guo, Xiongkang Gan, Yanming Hu, Qiunuo Li, Ming Sun*, Xianghua Liu**

Supplementary Figure S1 to Supplementary Figure S8

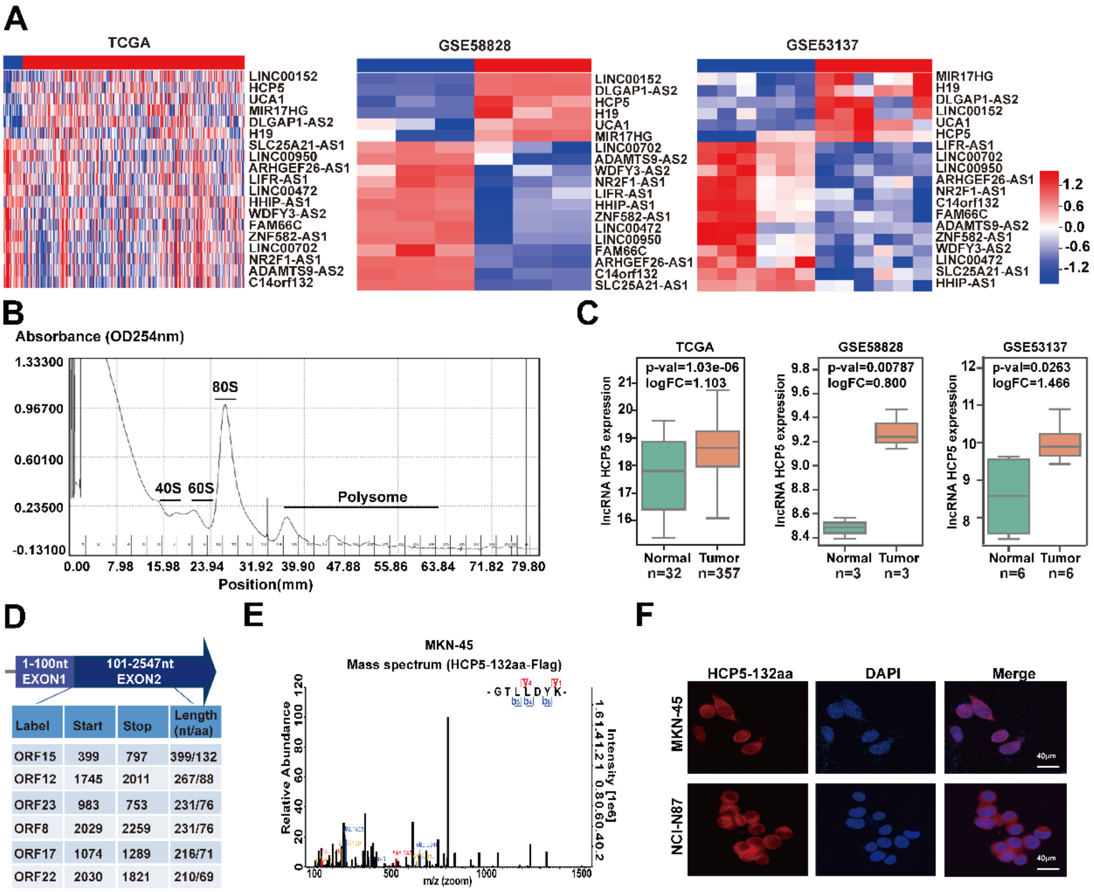

Label	Start	Stop	Length (nt/aa)
ORF15	399	797	399/132
ORF12	1745	2011	267/88
ORF23	983	753	231/76
ORF8	2029	2259	231/76
ORF17	1074	1289	216/71
ORF22	2030	1821	210/69

Figure S1. LncRNA HCP5 is upregulated in GC and has coding potential. A) The TCGA and GEO databases (GSE58828, GSE53137) were analyzed to identify the lncRNAs that are differentially expressed in GC tissues compared to normal stomach tissues. B) Polysome profiling was subjected to isolate the various components of ribosomes in GC cells. C) The expression of lncRNA HCP5 in GC and paraneoplastic tissue from TCGA and GEO databases (GSE58828, GSE53137). D) Genomic locations of six predicted ORFs of lncRNA HCP5 by ORF Finder. E) The unique HCP5-132aa-Flag peptide was identified using MS. F) Endogenous expression of HCP5-132aa was detected in GC cells by immunofluorescence staining using anti-HCP5-132aa antibody, scale bars, 40 μm.

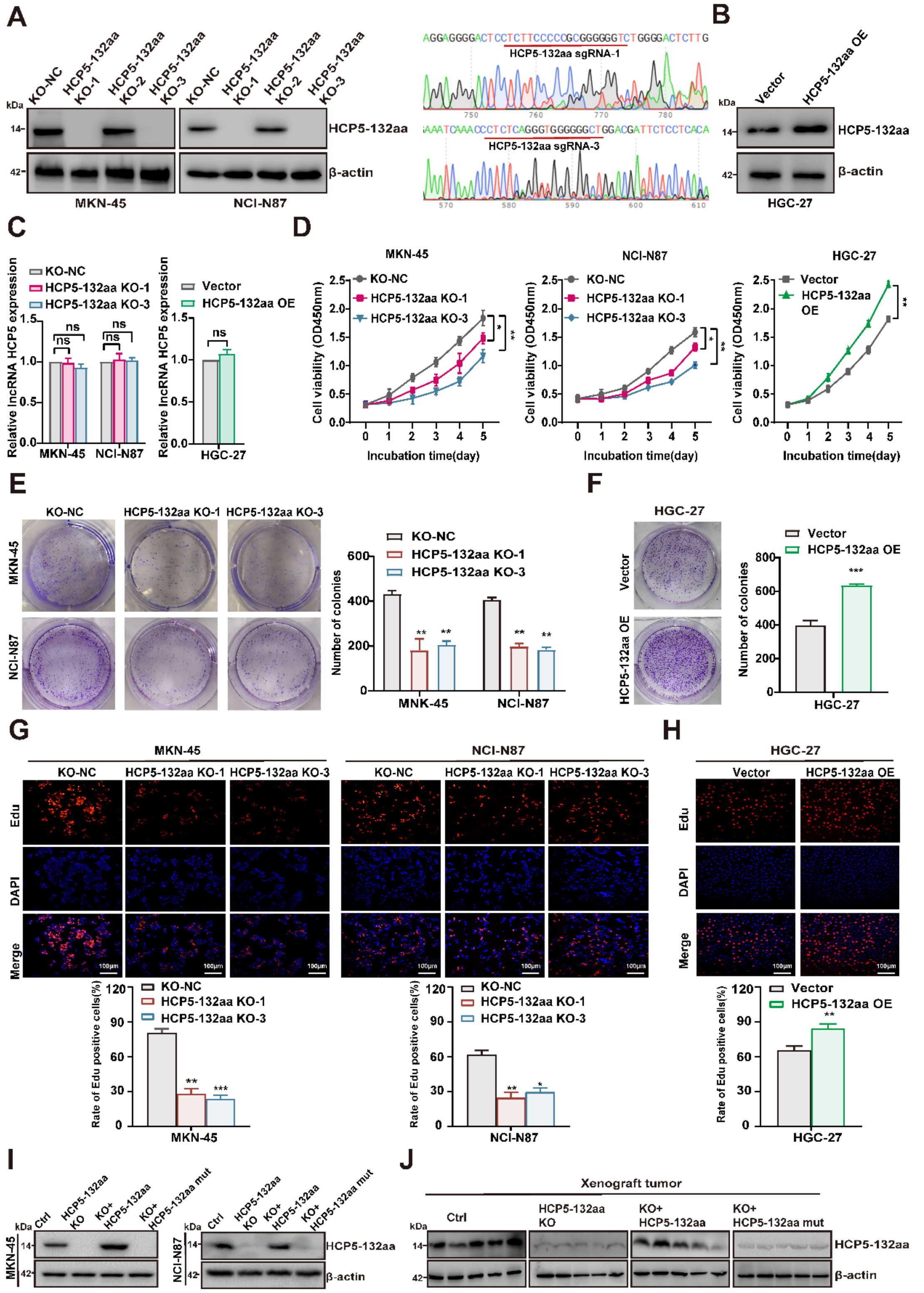

Figure S2. HCP5-132aa promotes the proliferation of GC cells. A, B) The knockout efficiency of HCP5-132aa in GC cell lines targeted by CRISPR/Cas9 system was determined by western blotting (left), and sanger sequencing showed that sgRNAs designed for the ORF15 region of HCP5-132aa successfully targeted the HCP5 genome (right). B) The stable overexpression efficiency of

HCP5-132aa in GC cells was detected by western blotting. C）The RNA expression level of lncRNA HCP5 was assessed by RT-qPCR (n=3) in GC cells with stable knockout or overexpression of HCP5-132aa. D-H) CCK-8 (D) (n=5), colony formation (E, F) (n=3), and Edu staining assay (G, H) (n=3) to detect the proliferative activity of GC cells with HCP5-132aa stable knockout or overexpression. I) The protein expression of HCP5-132aa was detected by western blotting in GC cells transfected with Ctrl, HCP5-132aa KO, KO+ HCP5-132aa and KO+HCP5-132aa mut. J) western blotting was performed to detect the protein expression levels of HCP5-132aa in the four groups of xenografts tumor tissues: Ctrl, HCP5-132aa KO, KO+HCP5-132aa and KO+HCP5-132aa mut. Data are represented as mean ± SD. Differences between the groups were evaluated using Student's t-test, ns indicates no significance, $*P < 0.05$, $**P < 0.01$, $***P < 0.001$.

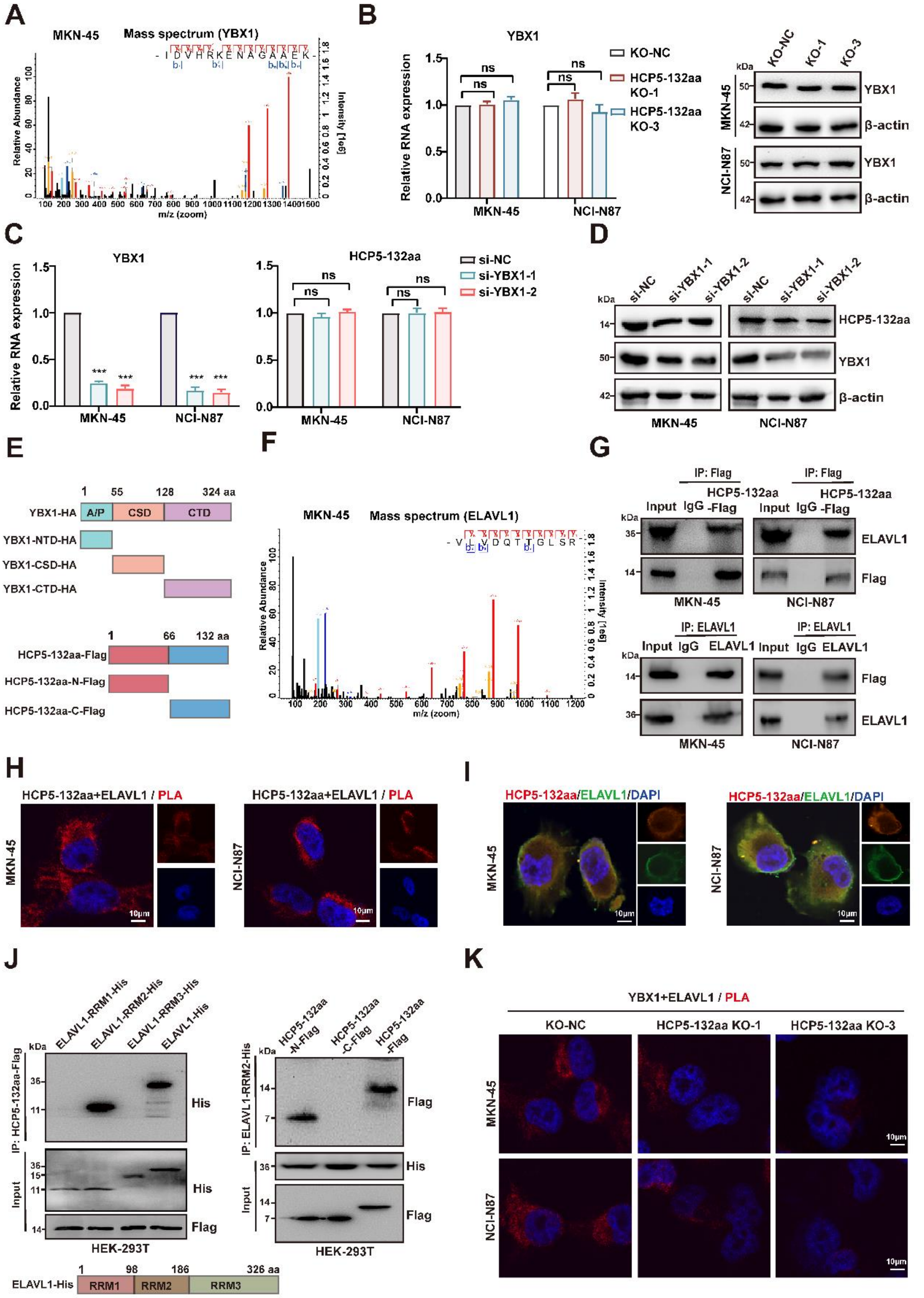

Figure S3. HCP5-132aa does not influence the mRNA and protein expression of YBX1, but rather interacts with its binding partner protein ELAVL1 in GC. A）The YBX1 unique peptides were identified using MS. B） The RNA and protein expression levels of YBX1 were evaluated by RT-qPCR (n=3) and western blotting in HCP5-132aa stable knockout GC cells. C, D) The RNA and

protein expression levels of YBX1 and HCP5-132aa were evaluated by RT-qPCR (n=3) and western blotting in YBX1-depleted GC cells. E) Schematic diagrams are presented to illustrate the wild-type HCP5-132aa and YBX1, as well as their truncation mutants. F) The ELAVL1 unique peptides were identified using MS. G, H) Co-IP and PLA assay were used to examine how HCP5-132aa and ELAVL1 interacted in GC cells, scale bar: 10μm. I) Immunofluorescence staining revealed the co-localization of HCP5-132aa and ELAVL1 in GC cells, scale bar: 10μm. J) The wild-type HCP5-132aa and ELAVL1, together with their corresponding truncation mutants, are illustrated in schematic diagrams. The indicated ELAVL1-His mutants were co-transfected with the HCP5-132aa-Flag vector into HEK-293T cells, followed by Co-IP with an anti-His antibody (left). The interaction of HCP5-132aa-Flag mutant with ELAVL1-RRM2-His were detected by Co-IP using anti-Flag antibodies (right). K) The interaction between YBX1 and ELAVL1 was detected in HCP5-132aa knockout GC cells utilizing PLA assay, scale bar: 10μm. The data are represented as the means ± SD. Differences between the groups were evaluated using Student's t-test, ns indicates no significance, ***p < 0.001.

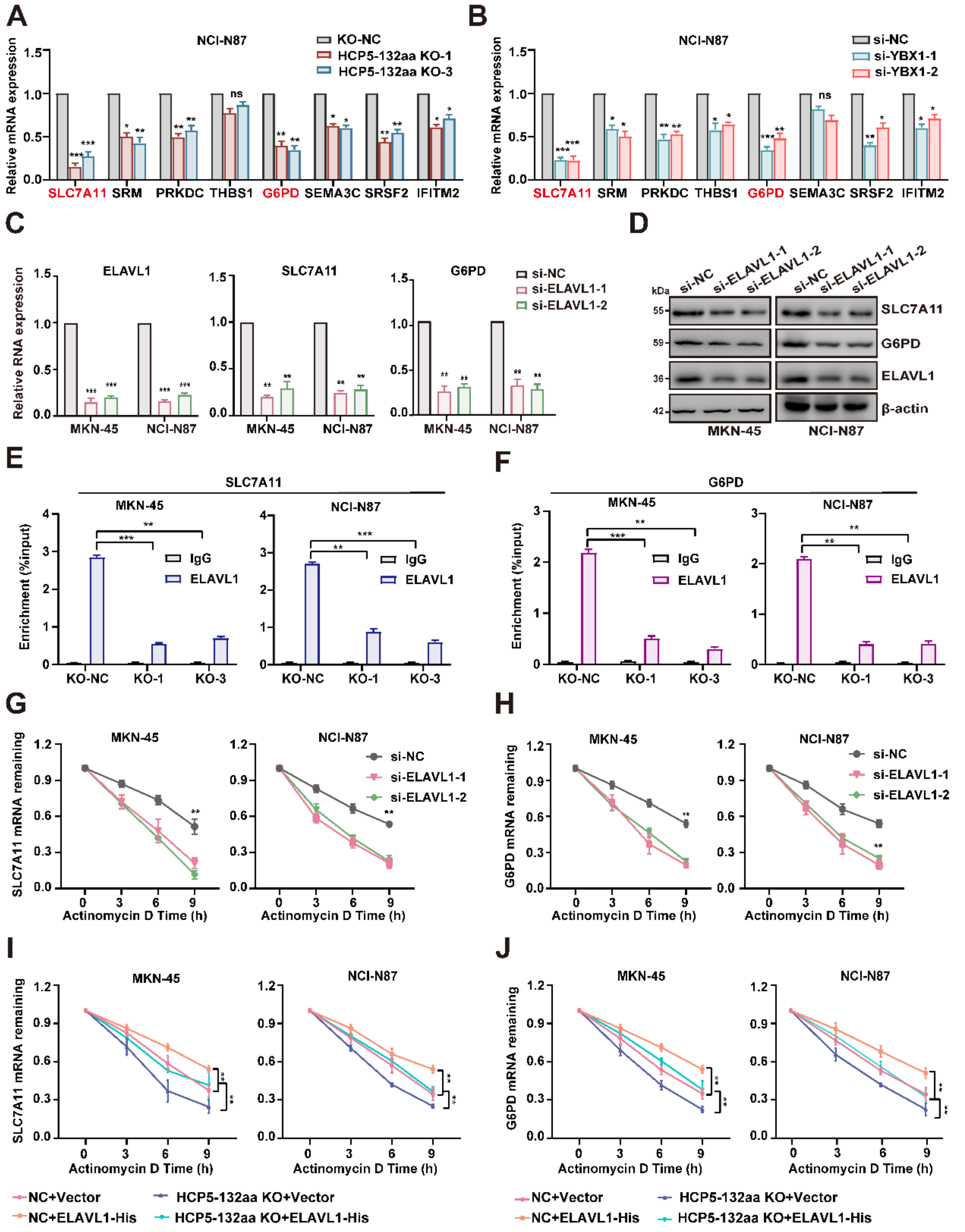

Figure S4. HCP5-132aa mediates ELAVL1 regulation of SLC7A11 and G6PD mRNA stability. A, B) The mRNA expression levels of 8 candidate target genes were evaluated by RT-qPCR in HCP5-132aa knockout or YBX1 knockdown GC cells (n=3). C, D) The mRNA and protein expression levels of ELAVL1, SLC7A11 and G6PD were evaluated by qPCR and western blotting in ELAVL1-depleted GC cells (n=3). E, F) The binding of ELAVL1 on SLC7A11or G6PD mRNA was determined in GC cells with HCP5-132aa knockout by an RIP-qPCR assay (n=3). G, H)

Following treatment with ActD, RT-qPCR analysis was conducted to determine the mRNA half-life of SLC7A11 and G6PD in GC cells in which ELAVL1 were knocked down (n=3). I, J) The increase in SLC7A11 and G6PD mRNA half-life caused by ELAVL1 expression was inhibited by the knockout of HCP5-132aa (n=3). Data are represented as mean ± SD. Differences between the groups were evaluated using Student's t-test (A-C, E-H) or One-way ANOVA with Tukey's Multiple Comparison test (I, J), ns indicates no significance, *P <0 .05, **P <0 .01, ***P < 0.001.

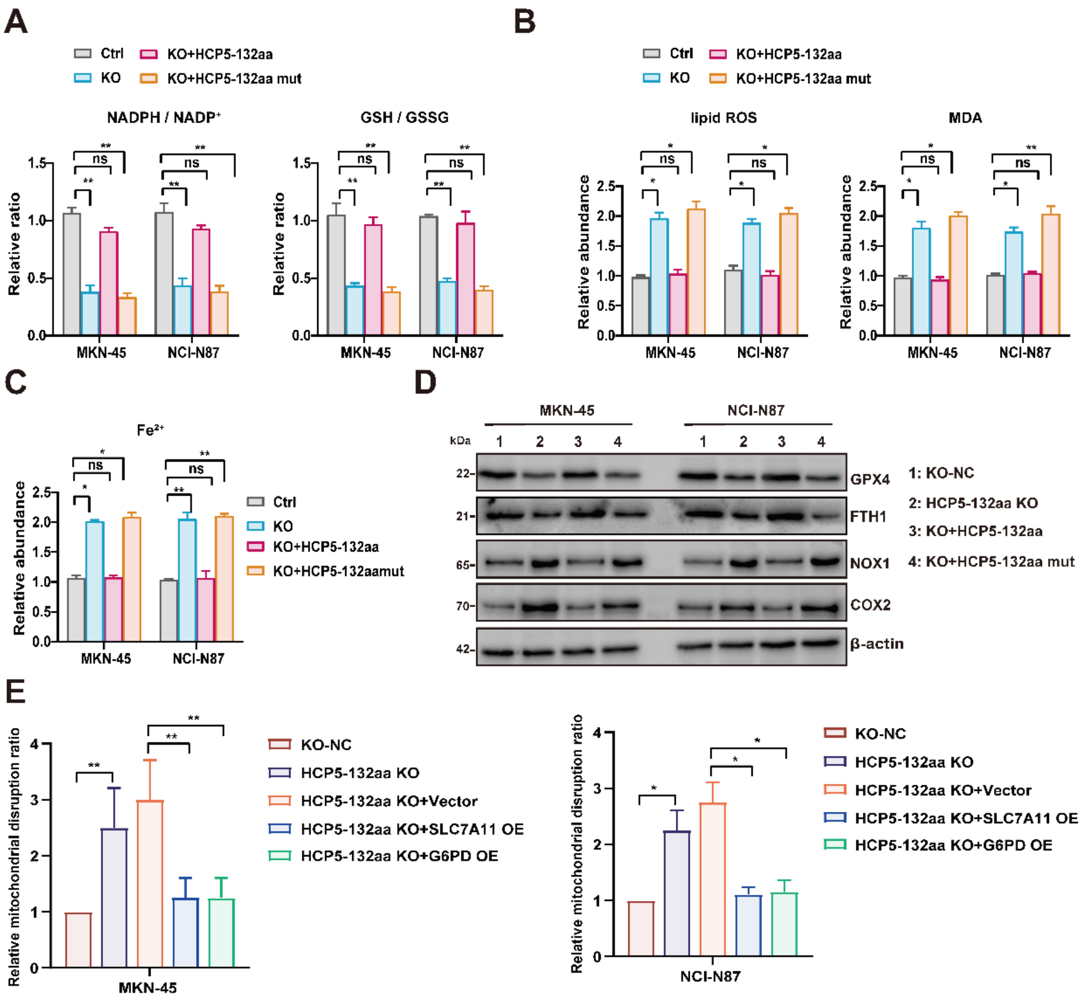

Figure S5. HCP5-132aa can regulate the occurrence of ferroptosis in GC cells. A, B) For the ferroptosis occurrence in the stable GC cell lines of Ctrl, HCP5-132aa KO, KO+HCP5-132aa and KO+ HCP5-132aa mut, were used for Coenzyme II NADP(H) Assay and GSH Assay kits (A) as well as the MDA assay kits and C11-BODIPY fluorescent probe (B) were used for detection. C) Intracellular Fe^{2+} in GC cells of different groups was analyzed by FerroOrange (n=3). D) The expression levels of ferroptosis-regulatory proteins, including GPX4, FTH1, NOX1, and COX2, were analyzed by western blotting in the above indicated GC cells. E) Quantification of disrupted

mitochondria in different groups of GC cells, including: KO-NC、HCP5-132aa KO、HCP5-132aa KO+Vector、HCP5-132aa KO+SLC7A11 OE、HCP5-132aa KO+G6PD OE. The data are shown as the mean ± SD. Differences between the groups were evaluated using One-way ANOVA with Tukey's Multiple Comparison test, ns indicates no significance, *P < 0.05, **P < 0.01.

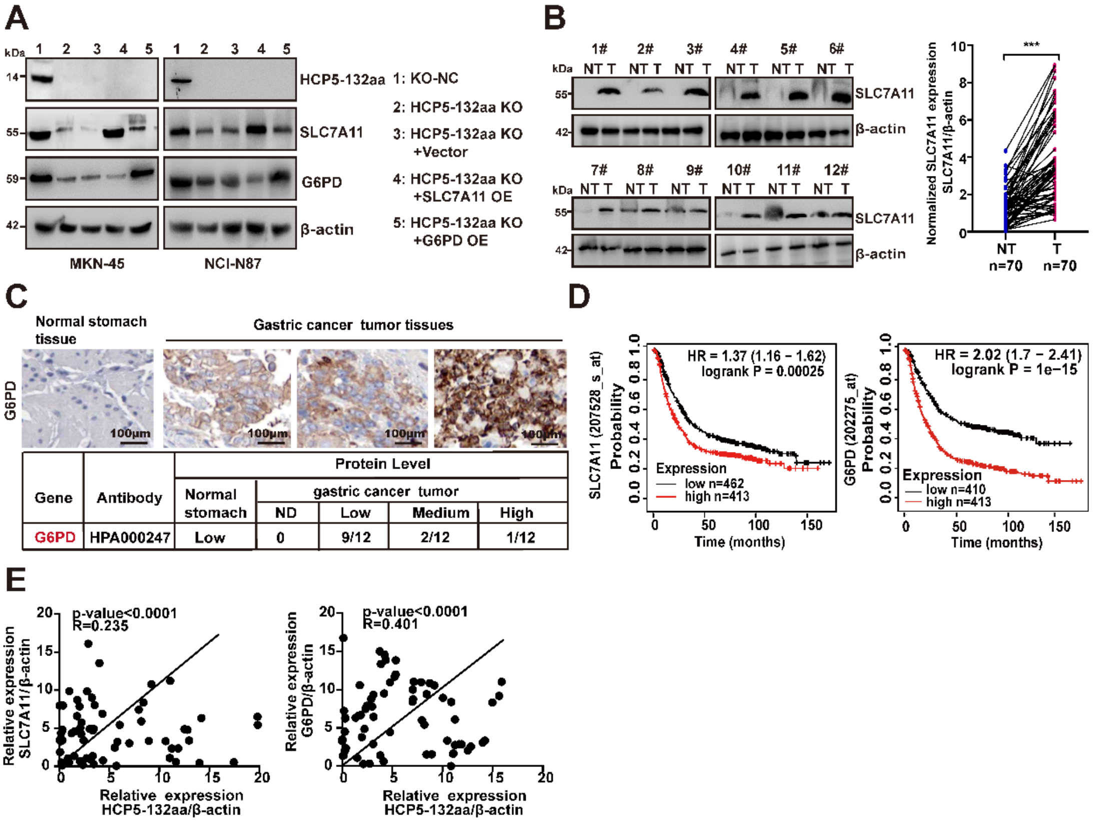

Figure S6. SLC7A11 and G6PD were highly expressed in GC, showing a strong correlation with HCP5-132aa protein expression. A) After transfection, protein expression of HCP5-132aa, SLC7A11, and G6PD was analyzed in GC cells with knocked out HCP5-132aa using western blotting. B) The expression levels of the SLC7A11 protein were detected using western blotting in 70 paired GC tissues (showing 12 pairs, left). Paired t-tests were used to compare expression levels of SLC7A11 protein (right). C) Analyze the IHC data in the HPA database to show the expression level of G6PD in GC tissues. D) Perform a Kaplan-Meier Plotter analysis to investigate the correlation between the expression levels of SLC7A11 and G6PD and the overall survival of GC patients. E) Correlation analysis was conducted to investigate the relationship between the relative abundances of HCP5-132aa and SLC7A11 or G6PD protein in 70 paired GC tissues. ***P < 0.001

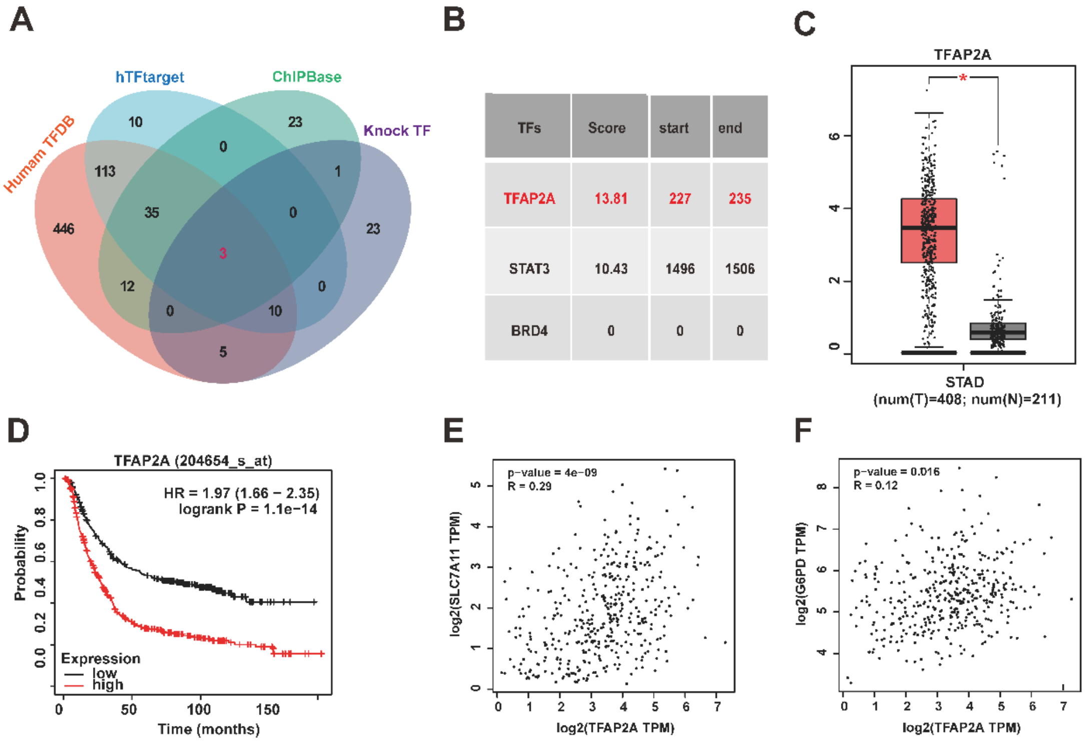

Figure S7. TFAP2A demonstrates a trend of high expression in GC, with the potential to function as an oncogene. A) The databases hTFtarget, Human TFDB, Knock TF, and ChIPBase collectively predict that three transcription factors, namely TFAP2A, STAT3, and BRD4, regulate the expression of lncRNA HCP5 in GC. B) JASPAR predicts the potential binding of these three transcription factors to the lncRNA HCP5 promoter region and ranks them based on scores. C) The GEPIA database analysis indicates high expression of TFAP2A in GC. D) Kaplan-Meier analysis demonstrates that patients with elevated expression levels of TFAP2A in GC have a poorer prognosis. E, F) The analysis from the GEPIA database reveals a positive correlation between the expression of TFAP2A in GC and SLC7A11 or G6PD. *P <0.05.

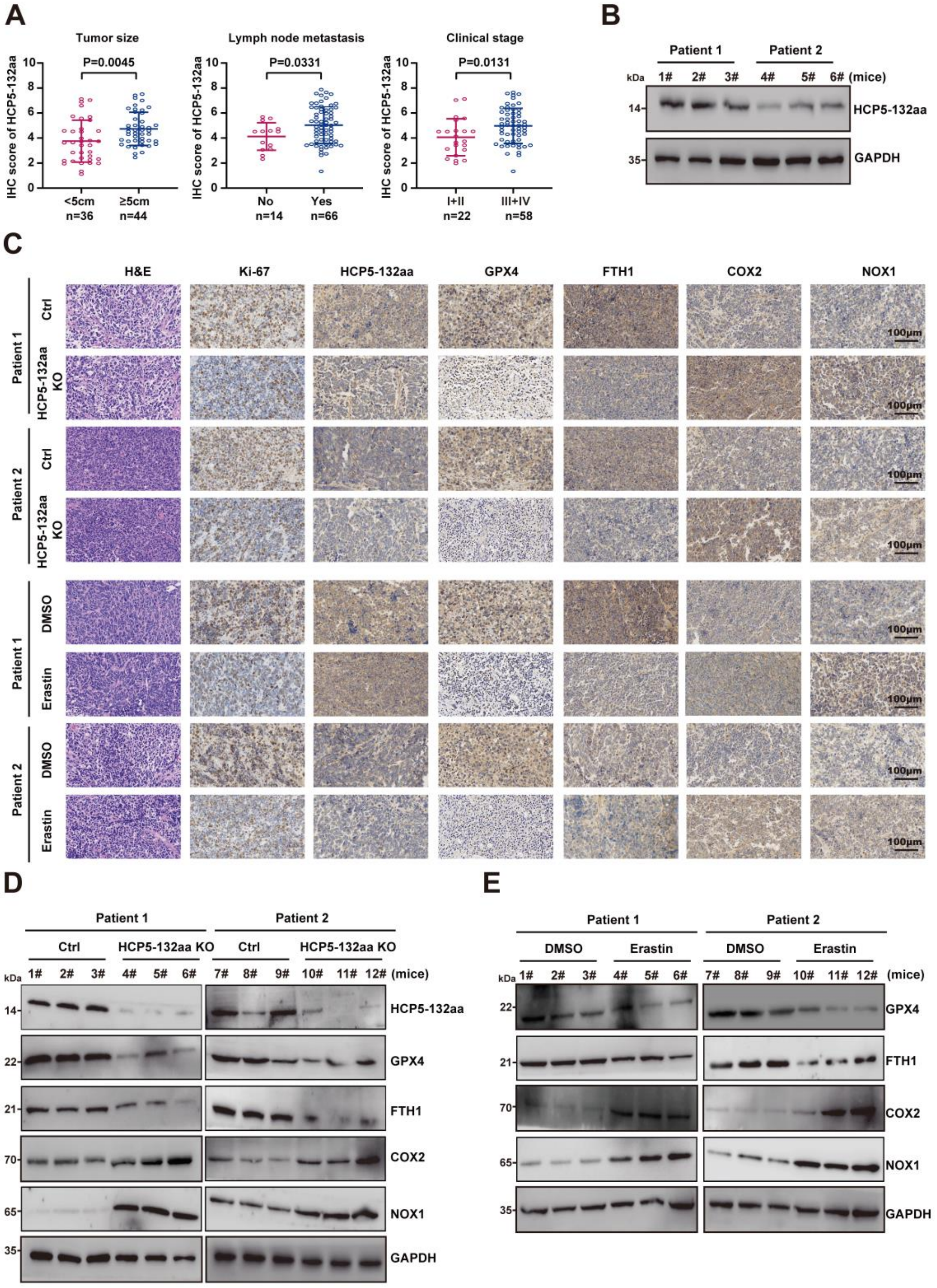

Figure S8. The HCP5-132aa has the potential to serve as a therapeutic target for GC. A). Correlation analysis of HCP5-132aa expression levels with tumor size, clinical stage and lymph node metastasis in 80 paired GC tumor microarrays. paired t test. B) Western blotting analysis revealed differential expression of HCP5-132aa in PDX models constructed from tumor tissues of two GC patients. C) The representative images of immunohistochemical evaluation of ki-67, HCP5-

132aa and ferroptosis markers expression in each group of GC-PDX tumor tissues, scale bars: 100 μm. D, E) Western blotting was performed to detect the protein levels of HCP5-132aa and ferroptosis markers in each group of PDX models (D), and also to assess the protein levels of ferroptosis markers in each group of PDX models treated with erastin compared to untreated controls (E). Data are represented as mean ± SD.

Supplementary Table S1 to Table S5

Supplementary Table S1. Correlation of the expression of HCP5-132aa with clinicopathologic features in gastric cancer.

Variables	N (%)	HCP5-132aa [a]		p-value
		High	Low	
Gender				0.0230*
Male	53(66%)	37	16	
Female	27(34%)	16	11	
Age(years)				0.1242
<55	34(43%)	18	16	
≥55	46(57%)	35	11	
Tumor size(cm)				0.0045**
≥5	44(55%)	28	16	
<5	36(45%)	13	23	
Lymph node metastasis				0.0331*
NO	14(18%)	8	6	
Yes	66(82%)	20	46	
TNM stage				0.0131*
I+II	22(28%)	11	11	
III+IV	58(72%)	42	16	
Tumor differentiation				0.1930
High	30(37%)	16	14	
Low	50(63%)	37	13	

[a] Fold change (FC) (tumor tissues relative to normal tissues) is greater than or equal to 2.0 for high expression, and less than 2.0 for low expression.

*P<0.05，**P<0.01 was considered significant (Chi-square test between 2 groups)

Supplementary Table S2. The RT-PCR primers used in this study

Gene names	Primers Sequences
lncRNA HCP5	Forward: TCTCCTTCTGCCCATCACTTG
	Reverse: AACCCTCCTCCTGCTGTTCTC
GAPDH	Forward: ATGACATCAAGAAGGTGGTGAAGCAGG
	Reverse: GCGTCAAAGGTGGAGGAGTGGGT
YBX1	Forward: AAGGAGAAAAGGGTGCGGAG
	Reverse: CCTACGACGTGGATAGCGTC
ELAVL1	Forward: CGCCAACTTGTACATCAGCG
	Reverse: TAAACGCAACCCCTCTGGAC
SLC7A11	Forward: CGTCCTTTCAAGGTGCCACT
	Reverse: GGCAGATTGCCAAGATCTCAAG
G6PD	Forward: AAACGGTCGTACACTTCGGG
	Reverse: GGTAGTGGTCGATGCGGTAG
TFAP2A	Forward: GCTACACTGAGACTCCCGTC
	Reverse: GCGTGCGTGTTCCTTAATCC
DLGAP1-AS2	Forward: GCGCCTAAGAAATGCCTGT
	Reverse: AGCTGTTCATTCAGCCACGA
LINC00152	Forward: TGGGAATGGAGGGAAATAAA
	Reverse: CCAGGAACTGTGCTGTGAAG
UCA1	Forward: CTCTCCATTGGGTTCACCATTC
	Reverse: GCGGCAGGTCTTAAGAGATGAG
MIR17HG	Forward: GGCGTCCCGTCGTAGTAAAG
	Reverse: CATTGTGTCAGGAGTCAGTGTGTC
H19	Forward: CGGCCTTCCTGAACACCTTA
	Reverse: TCATGTTGTGGGTTCTGGGA
IFITM2	Forward: GTCACCATGAACCACATTGTGCAAAC
	Reverse: CCCCCAGCATAGCCACTTCC
SRSF2	Forward: CCACTCAGAGCTATGAGCTACG
	Reverse: ACTCCTTGGTGTAGCGGTCC
SEMA3C	Forward: CCAAATGGCTAAGTGAACCT
	Reverse: AGTACACCTTAGCATCATTTGG
SRM	Forward: GTGGTGGCCTATGCCTACTG
	Reverse: CTCCTGGAAGTTCGTGCTCG
PRKDC	Forward: CATGGAAGAAGATCCCCAGA
	Reverse: TGGGCACACCACTTTAACAA
THBS1	Forward: GGGGAGATAACGGTGTGTTTG
	Reverse: CGGGGATCAGGTTGGCATT
NSUN2	Forward: GGTATCCTGAAGAACTTGCC
	Reverse: ATCTTATGATGAGGCCGCA

Supplementary Table S3. Targeted sequences of siRNAs or sgRNAs and probe sequences of SLC7A11 or G6PD used in this study

siRNA	Sequences
si-YBX1-1	GGAUAUGGUUUCAUCAACATT
si-YBX1-2	GCAGACCGUAACCAUUAUATT
si-ELAVL1-1	TACCAGTTTCAATGGTCATAA
si-ELAVL1-2	GAGCGATCAACACGCTGAA
si-TFAP2A-1	CCUGCUCACAUCACUAGUATT
si-TFAP2A-2	GGAAGAUCUUUAAGAGAAATT
si-NSUN2-1	GAGATCCTCTTCTATGATCTT
si-NSUN2-2	CACGTGTTCACTAAACCCTATTT

sgRNA	Sequences
HCP5-132aa-1	GAGACCAGCGGGTGAGAAG
HCP5-132aa-2	GGCTGCTGTCACACAATGA
HCP5-132aa-3	CCAGCCACCCAACCTGAGA

Probes	Sequences
SLC7A11-m^5C	TACATTAAAGAAGAGTTTCTAGGGGC[m5]
	TACTGTTTATGAGACACATCCAGG
SLC7A11-C	TACATTAAAGAAGAGTTTCTAGGGGC
	TACTGTTTATGAGACACATCCAGG
G6PD-m^5C	CTCTGAGCCCTGGGCACCCACCTCC[m5C]
	ACCCCCGCCACGGCCACCCTCCTTC
G6PD-C	CTCTGAGCCCTGGGCACCCACCTCC
	ACCCCCGCCACGGCCACCCTCCTTC

Supplementary Table S4. Antibodies used in this study

Antigens	Manufacturers	Applications
β-actin mAb	A3854, Sigma-Aldrich	WB
HCP5-132aa mAb	ABclonal	WB/IF/IHC
YBX1 pAb	ab76149, Abcam	WB/IP/IF
ELAVL1 pAb	A19622, ABclonal	WB/IP/IF
SLC7A11 pAb	A2413, ABclonal	WB
G6PD pAb	T56841, Abmart	WB
NSUN2 pAb	A24132, ABclonal	WB
GPX4 pAb	A1933, ABclonal	WB
FTH1 pAb	A19544, ABclonal	WB
NOX1 pAb	A8527, ABclonal	WB
COX2 pAb	A1253, ABclonal	WB
DDDDK-tag pAb	AP0007, Biogot Technology	WB
DYKDDDDK-tag mAb	MA1-91878, Thermo Fisher Scientific	IP/IF
m^5C mAb	Ab10805, Abcam	MeRIP
GFP-tag mAb	AP0675M, Biogot Technology	WB/IP
HA-tag mAb	AP0005M, Biogot Technology	WB/IP
His-tag mAb	BS67270, Biogot Technology	WB/IP
GST-tag mAb	BS67272, Biogot Technology	WB/IP
ki-67(V3242) pAb	BS1454, Biogot Technology	IHC
HRP-linked anti-rabbit IgG	BS13278, Biogot Technology	WB
HRP-linked anti-Mouse IgG	BS12478, Biogot Technology	WB
Goat Anti-Rabbit IgG(H+L) Cy3	BS10007, Biogot Technology	IF
Goat Anti-Mouse IgG(H+L) 488	BS10015, Biogot Technology	IF

Supplementary Table S5. MS identified the top 30 scoring RNA-binding proteins that engage in specific interactions with HCP5-132aa.

UniProt ID	Gene names	Sequence coverage (%)	Unique Peptides	Score
Q9UGI8	TES	18.3	7	59.867
P50914	RPL14	35.3	5	56.952
P67809	YBX1	34.6	5	52.939
P61313	RPL15	25	4	44.249
P42704	LRPPRC	3.9	4	33.682
Q9UQ80	PA2G4	10.4	3	65.829
P13639	EEF2	5.7	3	30.534
Q13151	HNRNPA0	16.1	3	24.933
P04843	RPN1	8.9	3	22.567
O43390	HNRNPR	4.3	2	33.826
P30101	PDIA3	7.1	2	29.955
O43707	ACTN4	4.4	2	26.376
P61981	YWHAG	8.5	2	25.943
Q15717	ELAVL1	17.5	2	25.341
P23284	PPIB	10.2	1	23.469
P04843	RPN1	8.9	3	22.567
Q15365	PCBP1	5.9	1	18.618
Q6PKG0	LARP1	3.4	2	17.844
P16401	HIST1H1B	13.7	2	13.114
Q7Z2W4	ZC3HAV1	3.7	1	12.986
P05198	EIF2S1	10.2	2	12.581
Q9NVP1	DDX18	4	2	12.039
Q08J23	NSUN2	3.1	1	11.286
Q96QR8	PURB	4.2	1	10.408
Q86U42	PABPN1	5.2	1	8.927
Q9BVP2	GNL3	3.1	1	7.2354
P27824	CANX	2.5	1	7.2138

P62857	RPS28	18.8	1	7.1769
Q9NZI8	IGF2BP1	2.9	1	6.7338
Q9Y3I0	RTCB	2.8	1	6.5386
Q9H0D6	XRN2	1.3	1	6.4894
Q7KZF4	SND1	1.2	1	6.2588
P20042	EIF2S2	3	1	5.9941
Q2VIR3	EIF2S3B	11	3	100.2
P32969	RPL9	30.7	3	24.734
K7EM56	RPS15	11.6	1	12.077
P56192	MARS	2.3	2	11.611
H7BY36	EWSR1	4.5	1	6.1342
Q13148	TARDBP	7.4	1	6.1248

Uncropped blots of related figure

Figure 1

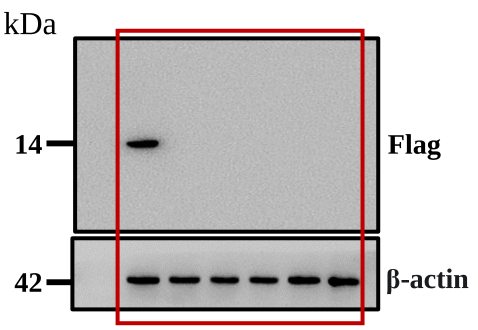

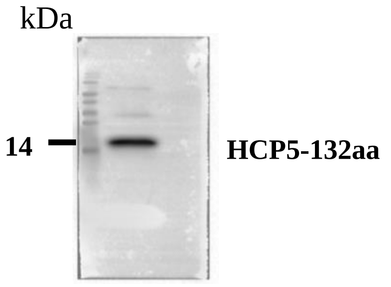

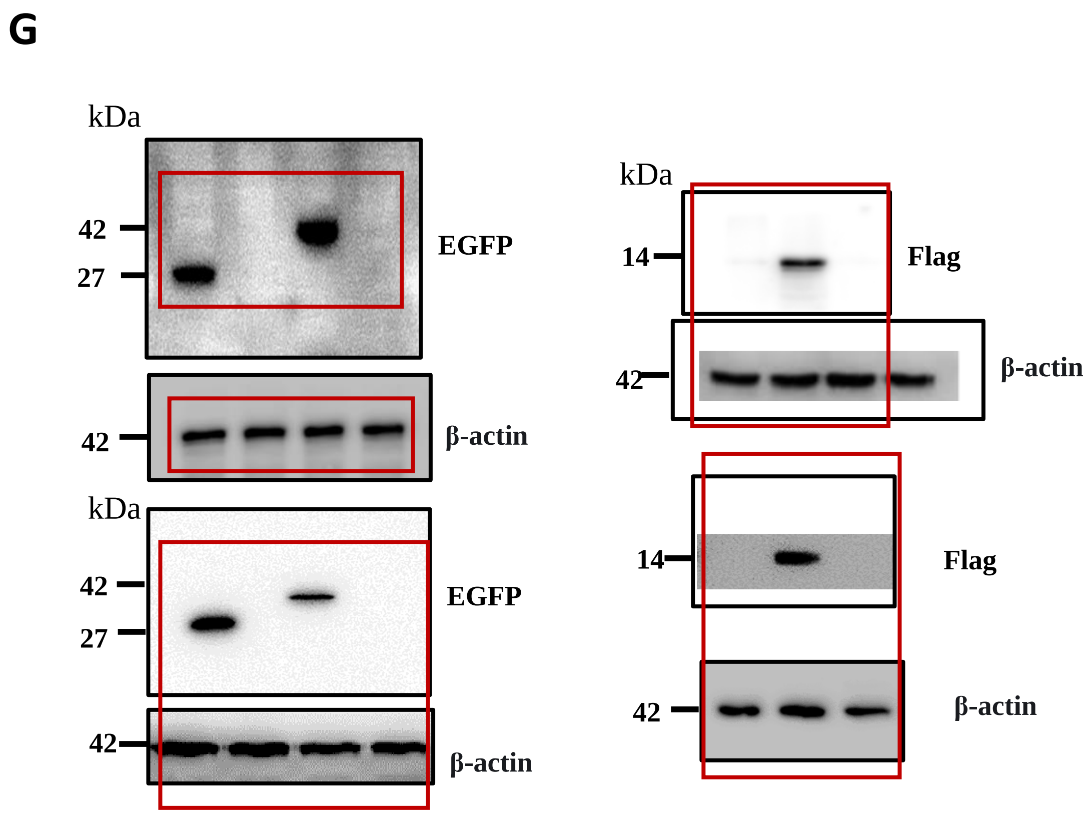

Figure 1

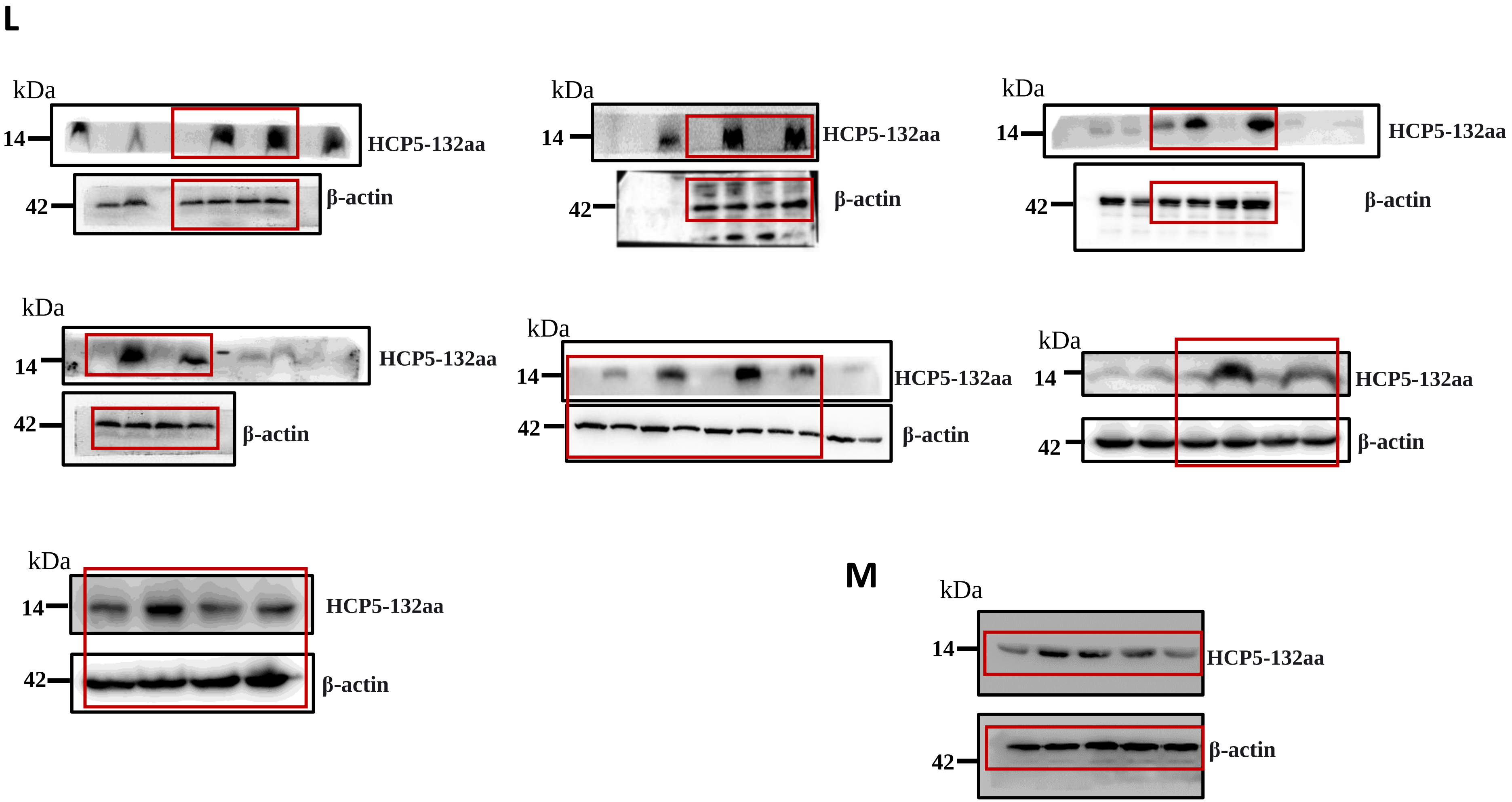

Figure 3

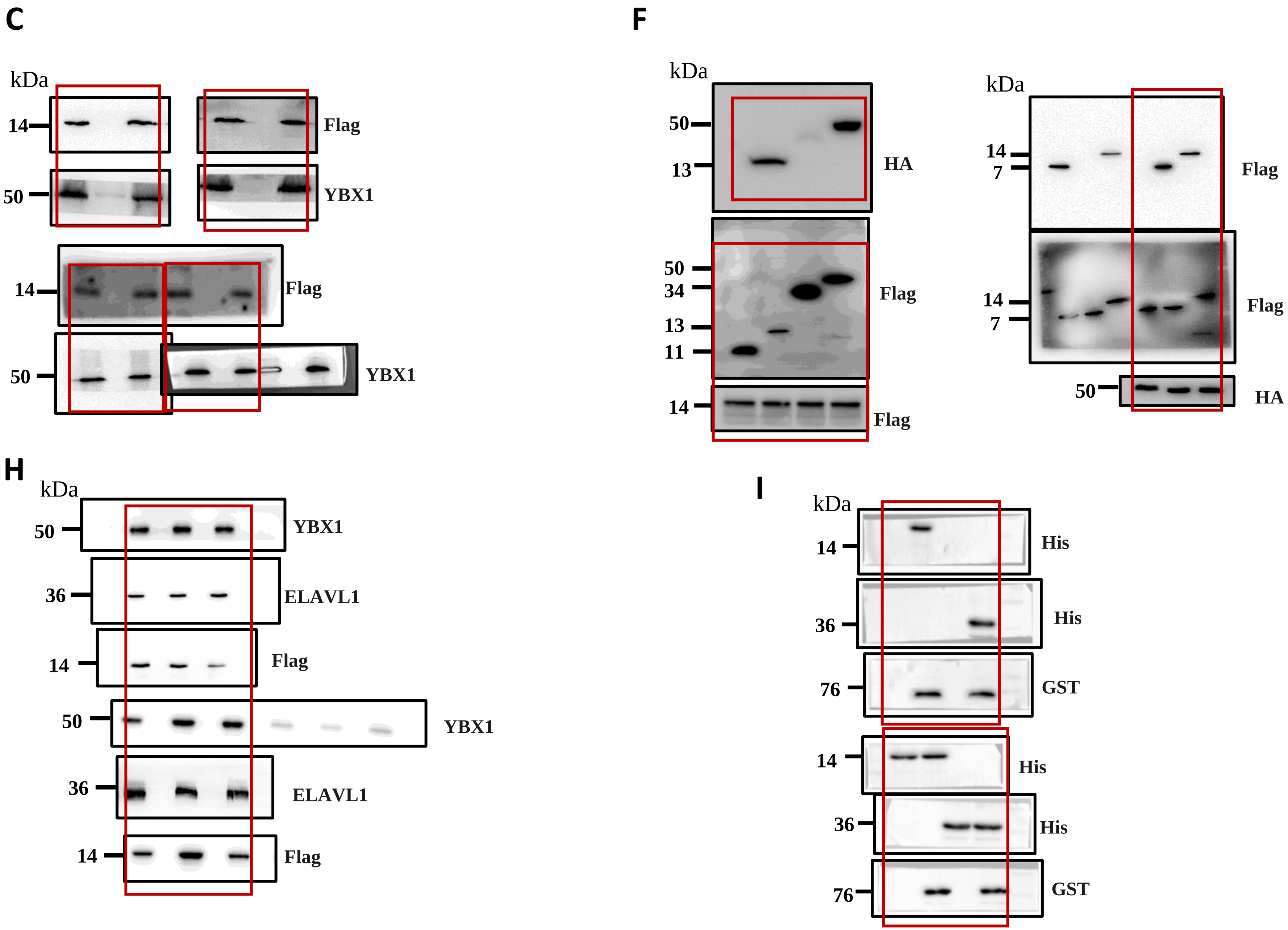
C
kDa
14
50
Flag
YBX1
14
Flag
50
YBX1
F
kDa
50
13
HA
50
34
13
11
Flag
14
Flag
kDa
14
7
Flag
14
7
Flag
50
HA
H
kDa
50
YBX1
36
ELAVL1
14
Flag
50
YBX1
36
ELAVL1
14
Flag
I
kDa
14
His
36
His
76
GST
14
His
36
His
76
GST

Figure 3

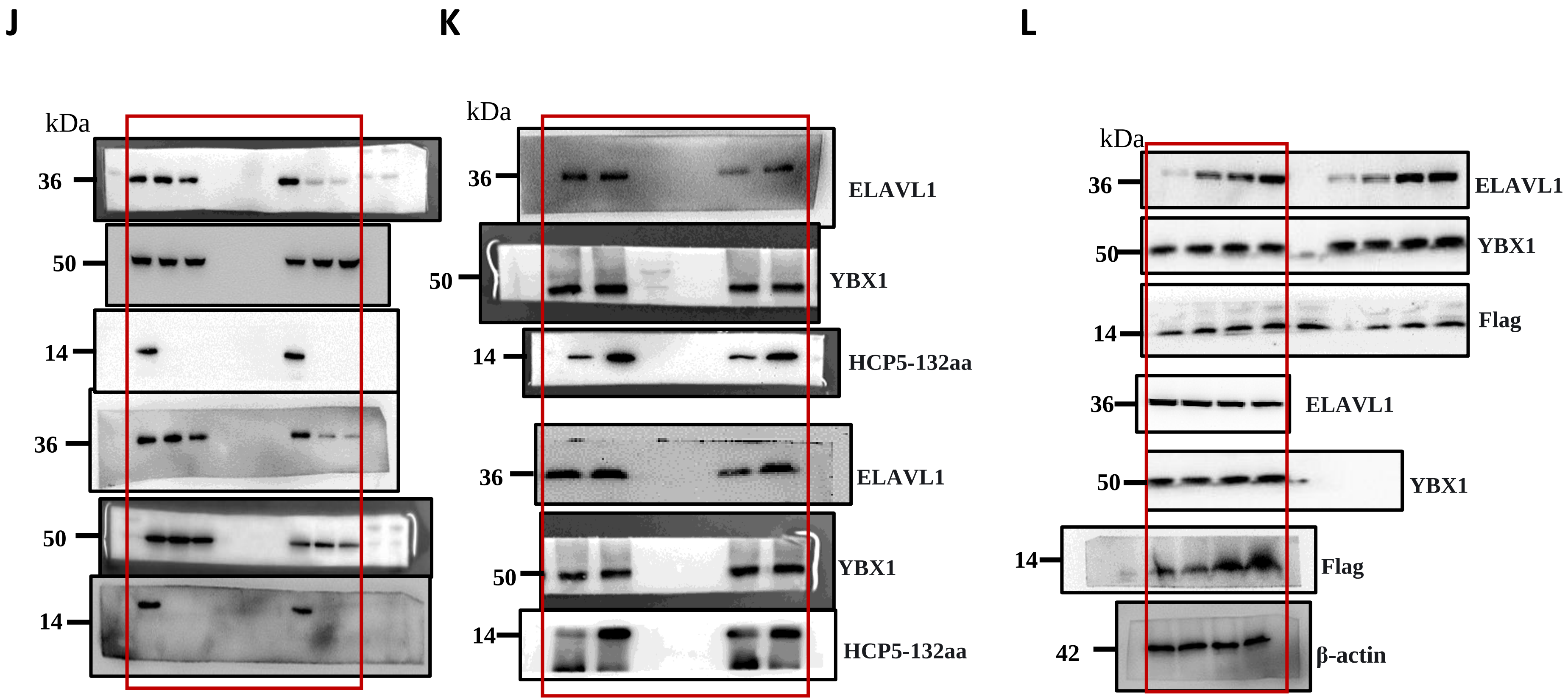
J
K
L
kDa
36
50
14
36
50
14
kDa
36
ELAVL1
50
YBX1
14
HCP5-132aa
36
ELAVL1
50
YBX1
14
HCP5-132aa
kDa
36
ELAVL1
50
YBX1
14
Flag
36
ELAVL1
50
YBX1
14
Flag
42
β-actin

Figure 4

Figure 5

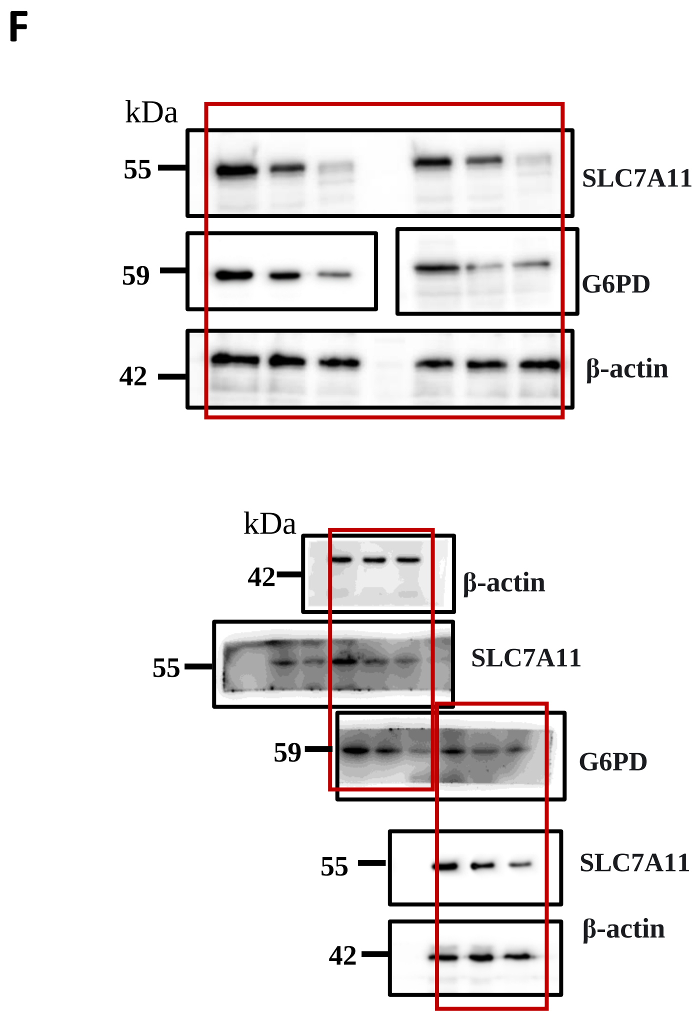

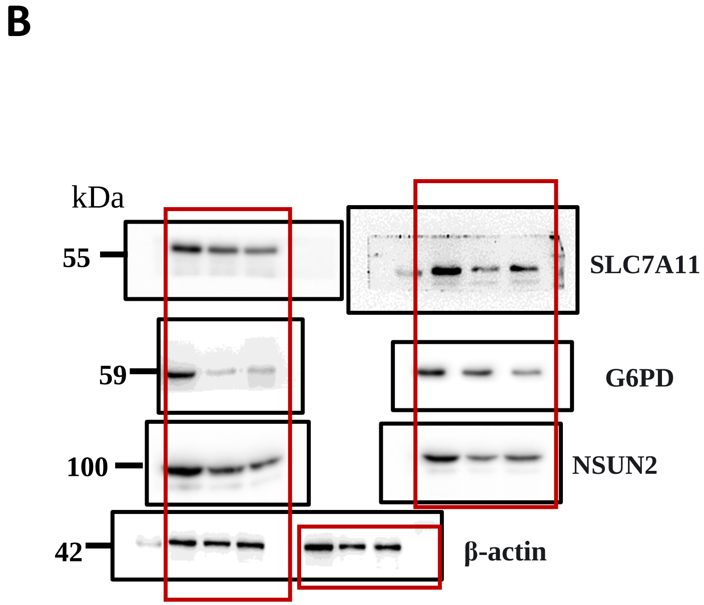

Figure 5

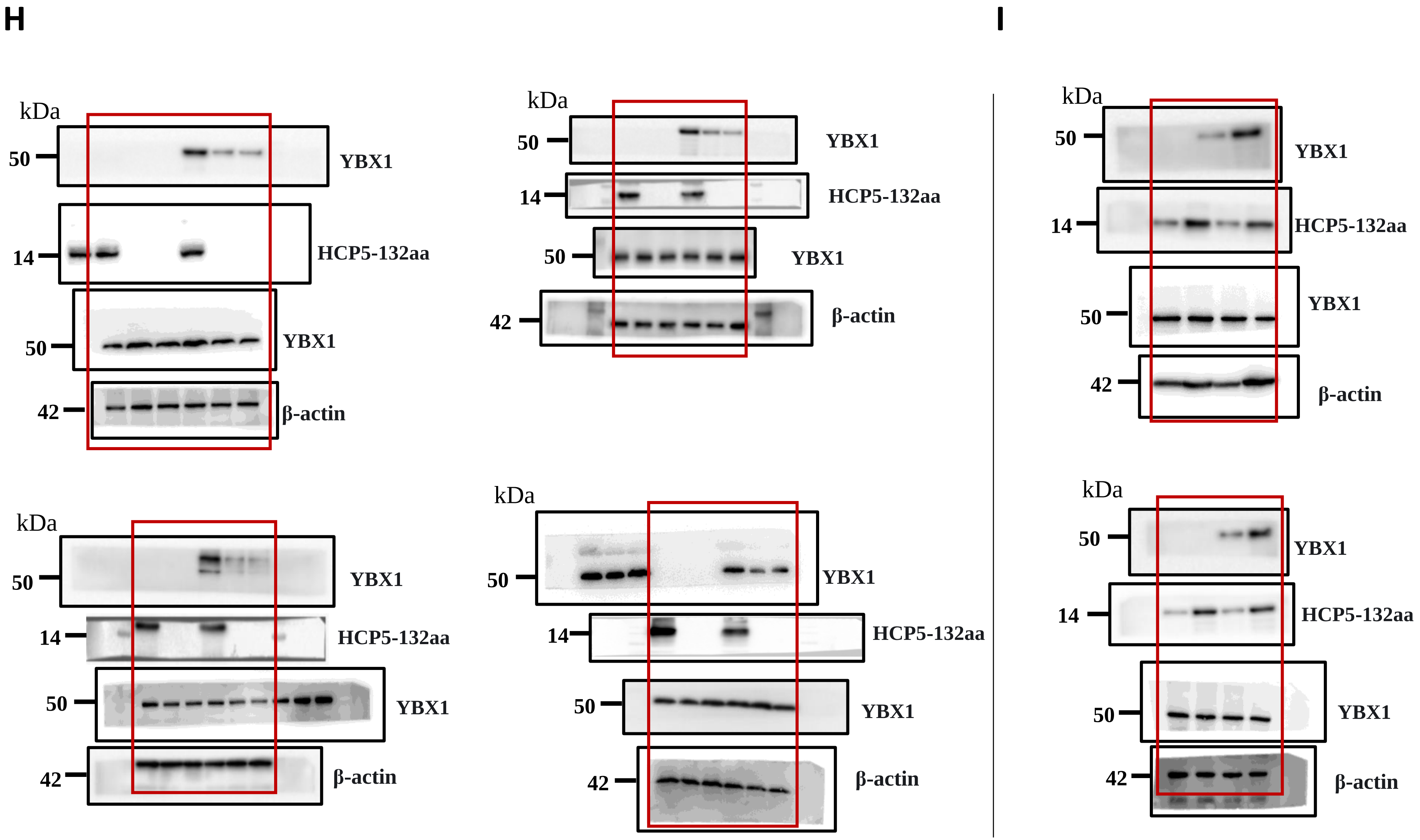
H
kDa
50
YBX1
14
HCP5-132aa
50
YBX1
42
β-actin
kDa
50
YBX1
14
HCP5-132aa
50
YBX1
42
β-actin
kDa
50
YBX1
14
HCP5-132aa
50
YBX1
42
β-actin
kDa
50
YBX1
14
HCP5-132aa
50
YBX1
42
β-actin
I
kDa
50
YBX1
14
HCP5-132aa
50
YBX1
42
β-actin
kDa
50
YBX1
14
HCP5-132aa
50
YBX1
42
β-actin

Figure 6

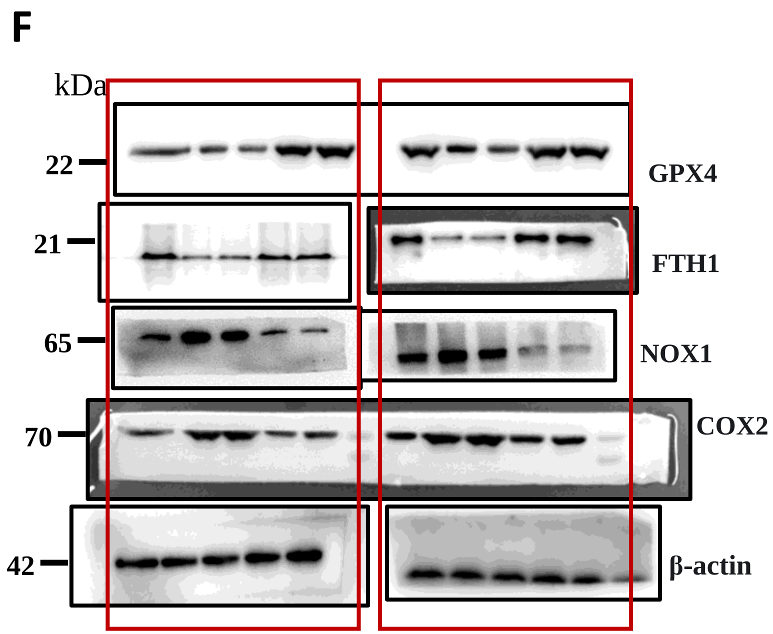

Figure 7

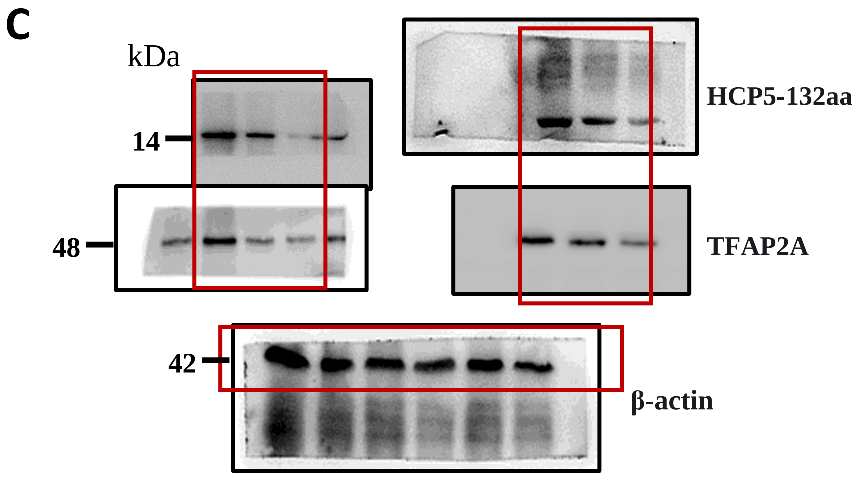

Figure S2

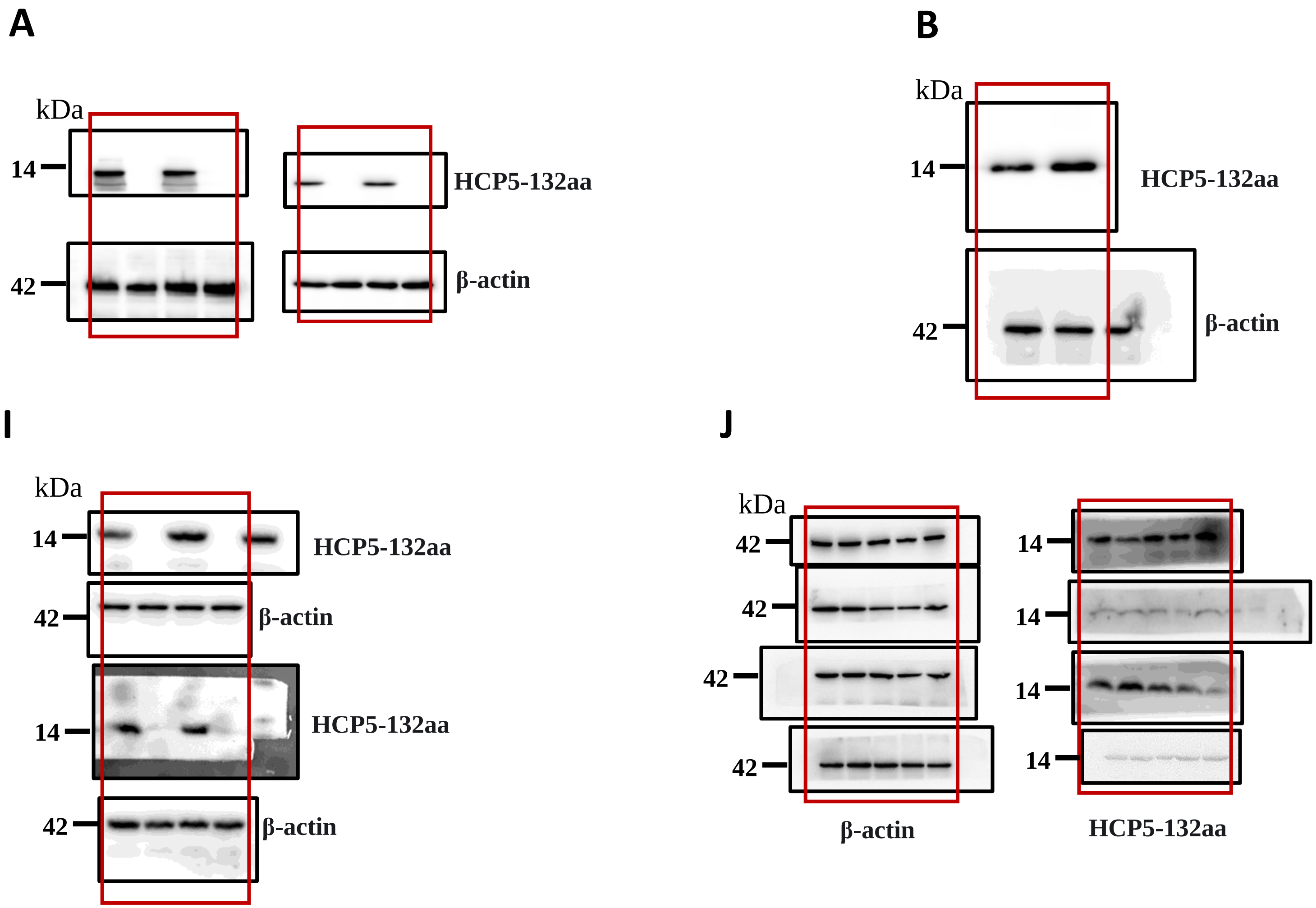
A
kDa
14
42
HCP5-132aa
β-actin
B
kDa
14
42
HCP5-132aa
β-actin
I
kDa
14
42
HCP5-132aa
β-actin
14
42
HCP5-132aa
β-actin
J
kDa
42
42
42
42
β-actin
14
14
14
14
HCP5-132aa

Figure S3

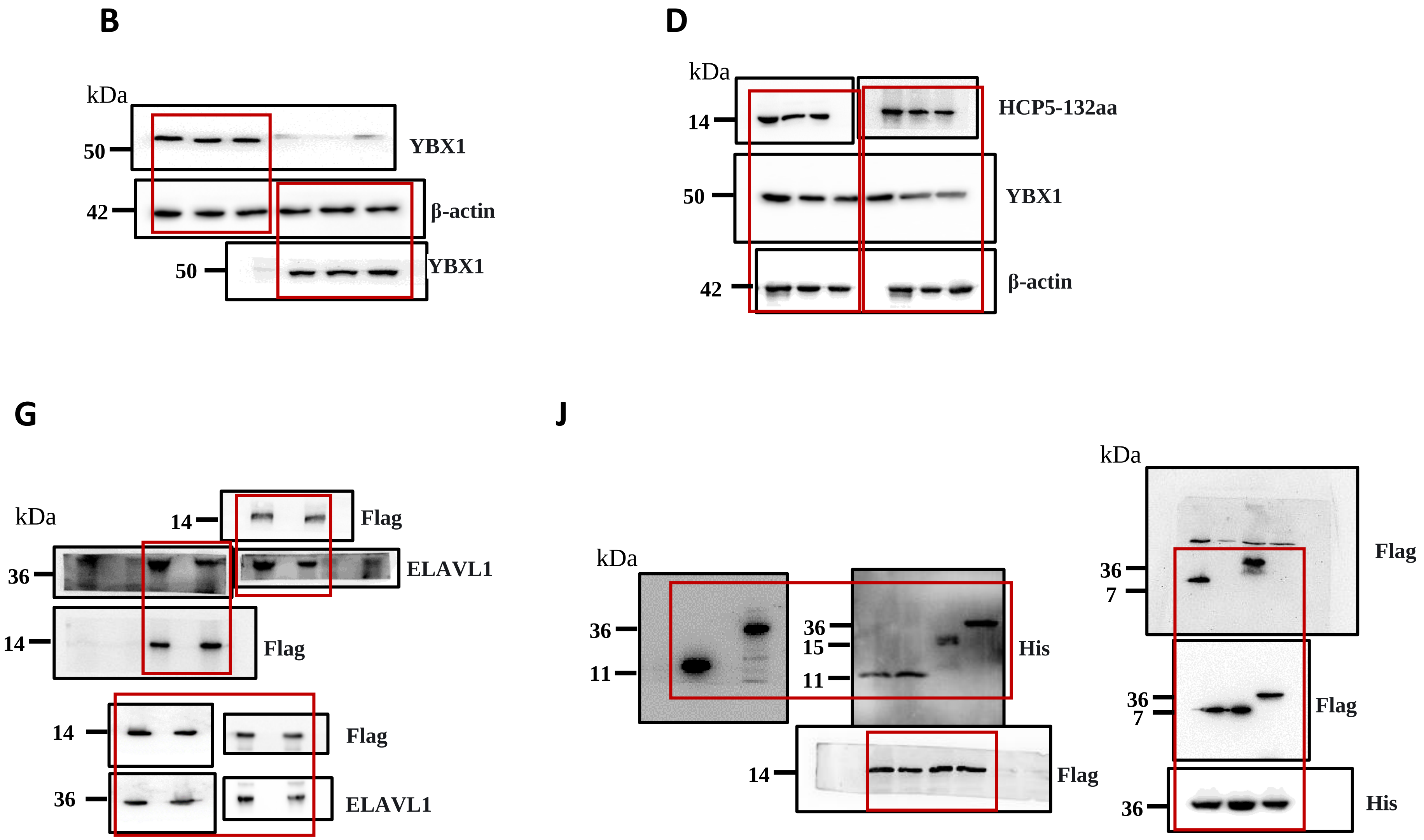

B
kDa
50
YBX1
42
β-actin
50
YBX1
D
kDa
14
HCP5-132aa
50
YBX1
42
β-actin
G
kDa
14
Flag
36
ELAVL1
14
Flag
14
Flag
36
ELAVL1
J
kDa
36
11
36
15
11
His
14
Flag
kDa
Flag
36
7
36
7
Flag
36
His

Figure S4

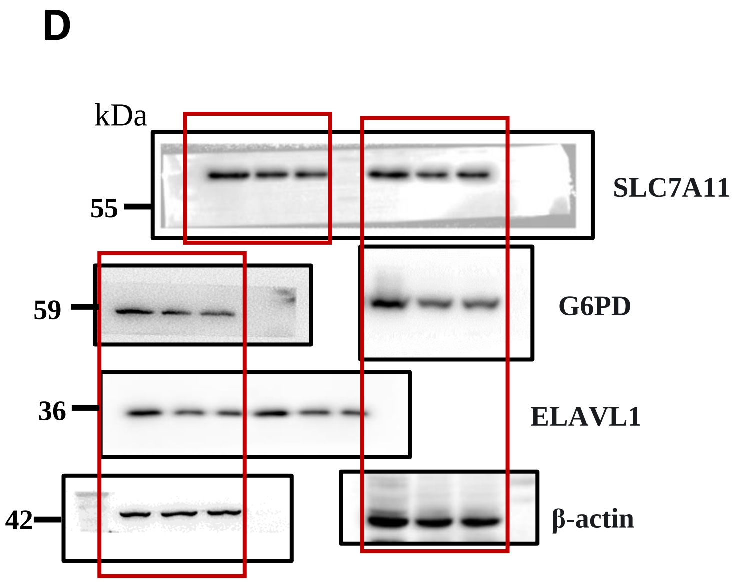

Figure S5

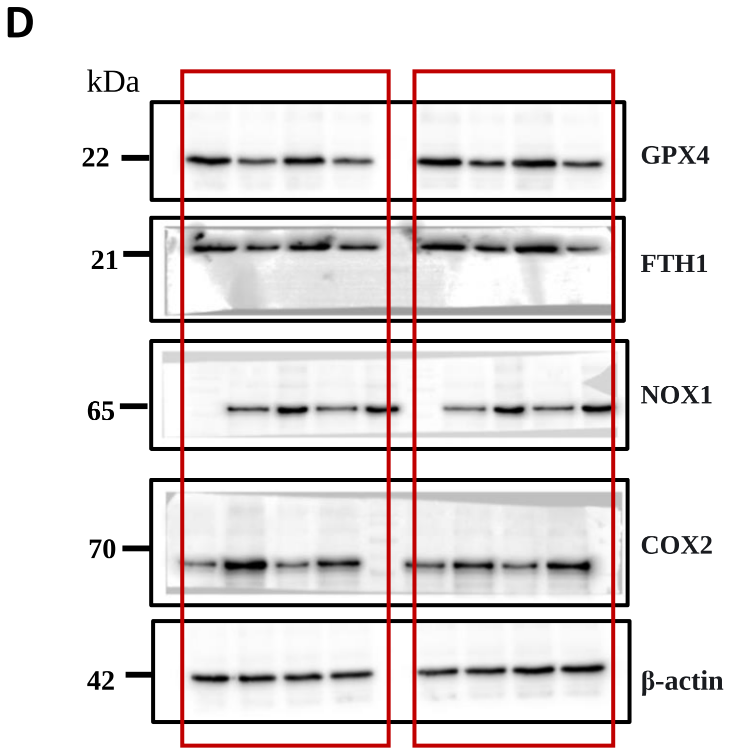

Figure S6

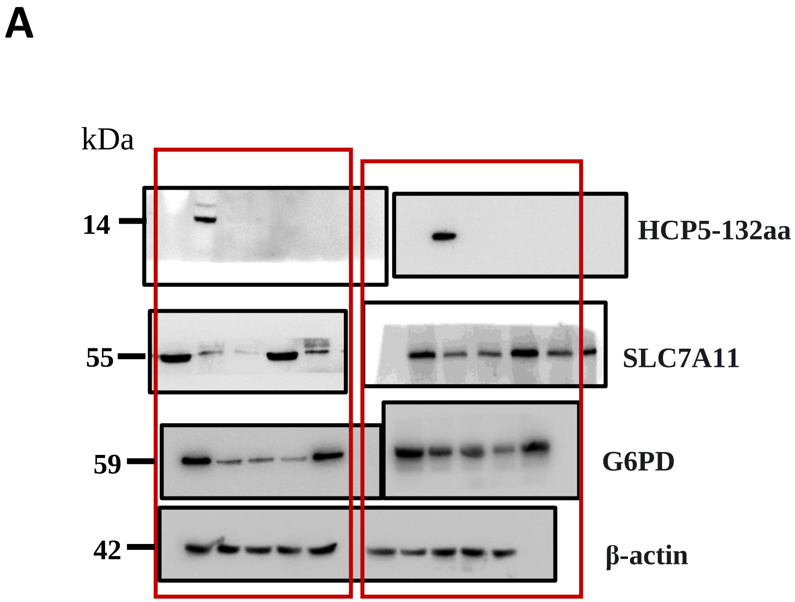
A
kDa
14
55
59
42
HCP5-132aa
SLC7A11
G6PD
β-actin

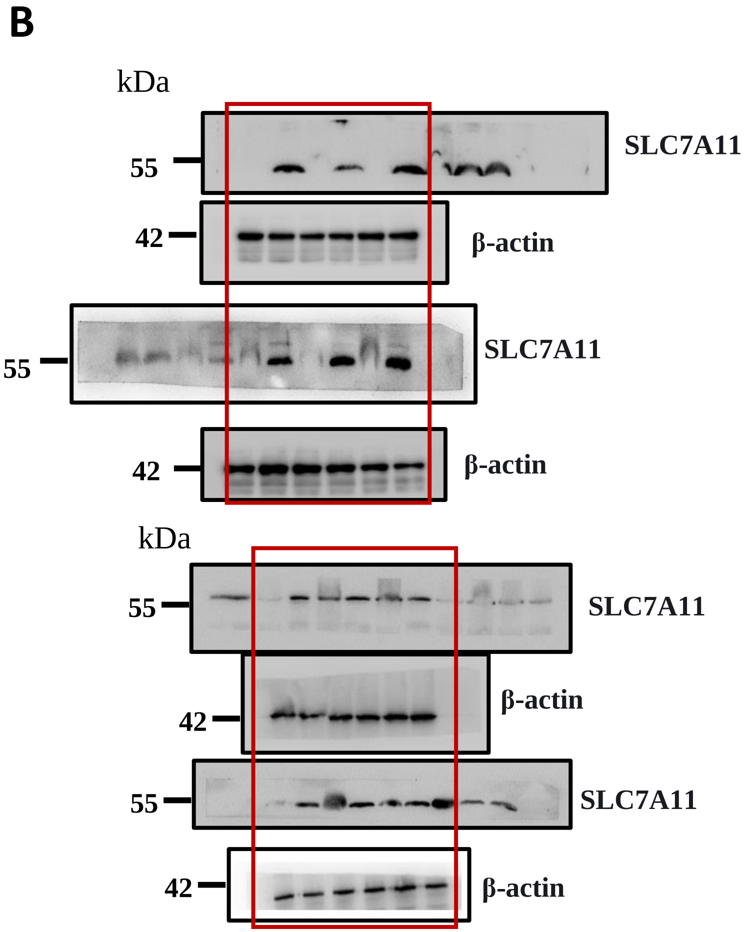
B
kDa
55
42
55
42
SLC7A11
β-actin
SLC7A11
β-actin
kDa
55
42
55
42
SLC7A11
β-actin
SLC7A11
β-actin

Figure S8

B

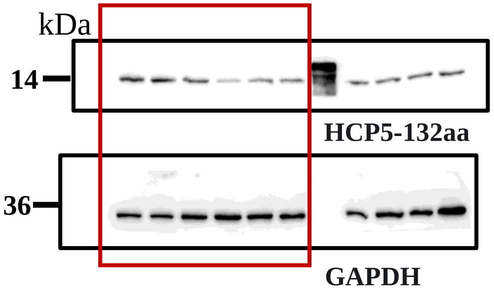

D

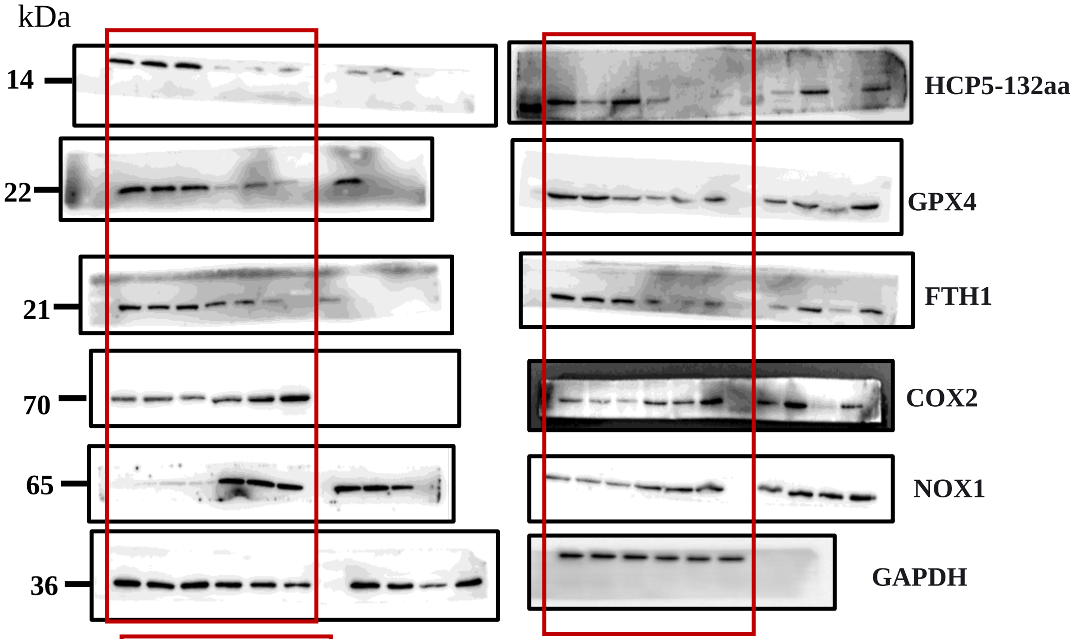

E

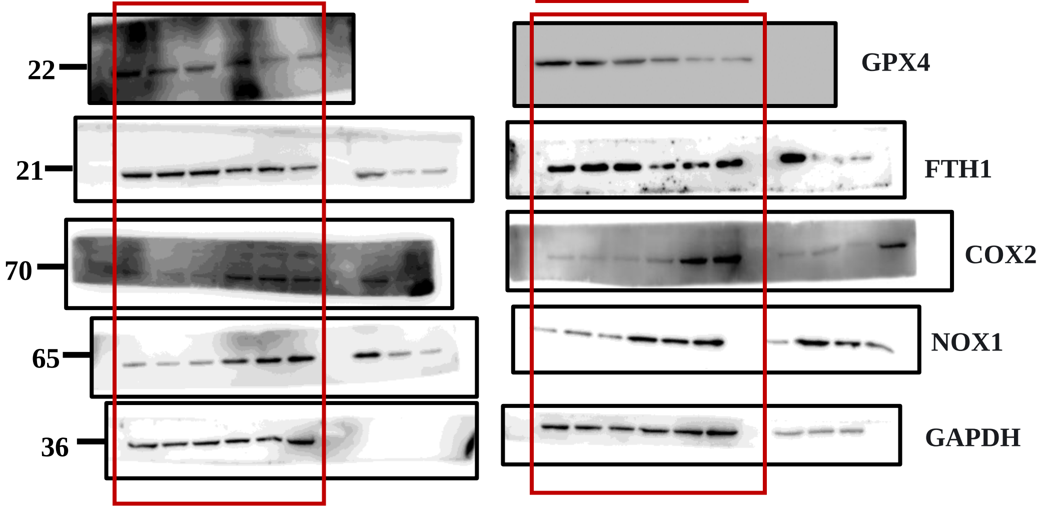

Supplement: Supplementary file 1 — Supporting Information [file ADVS-11-2407012-s001.pdf]
